# All roads lead to Rome: integrated physiological and transcriptomic analysis of cacao drought response reveals different ways to achieve tolerance in two hybrid clones

**DOI:** 10.3389/fpls.2026.1764400

**Published:** 2026-02-18

**Authors:** Mayra Andreina Osorio Zambrano, Loyla Rodríguez Pérez, Irene Papatheodorou, Wilson Terán

**Affiliations:** 1 Plant and Crop Biology, Department of Biology, Pontificia Universidad Javeriana, Bogotá, Colombia; 2 Gene Expression Team, European Molecular Biology Laboratory, European Bioinformatics Institute (EMBL-EBI), Wellcome Genome Campus, Hinxton, United Kingdom

**Keywords:** photosynthesis, RNA-Seq, *Theobroma cacao*, tolerance candidate genes, water deficit stress

## Abstract

**Introduction:**

Global warming poses significant challenges to agriculture through increased extreme weather events, such as the water deficit, affecting the establishment and yield of crops like cacao and all its value chain. Understanding the complex drought response mechanisms in cacao through integrated methodologies is crucial for developing strategies to enhance crop resilience to this stress.

**Methods:**

Here, we evaluated the response to a 52 days-long water deficit stress of three commercial cacao hybrid clones: EET8, ICS60 and TSH565 combining growth and physiological parameters with transcriptomic profiles.

**Results:**

TSH565 and EET8 clones exhibited the highest drought-stress tolerance through different strategies, being able to cope with stress and to better recovery after rewatering. TSH565 showed stomatal limitation but maintained unimpaired photosynthesis under drought. This clone also displayed water use efficiency and relative water content levels comparable to the watered control group, and its total dry weight exceeded that of EET8 and ICS60 under stress. Transcriptomic profiling of TSH565 indicated upregulation of genes encoding aquaporins, PSII proteins, proteins of the antioxidant system and several enzymes participating in the synthesis of osmo-protective secondary metabolites, seemingly contributing to its tolerance. In contrast, EET8 experienced both stomatal limitation and impaired photosynthetic machinery upon the same stress. Its higher stomatal conductance led to a concomitant increased water loss with a significant decrease in leaf water potential. Transcriptomic profiling revealed the activation of numerous biological processes and metabolic pathways, including key hub transcription factors probably responsible for inducing several downstream effector genes, ultimately driving to its stress tolerance. The induction of genes related to acclimation to low water potential and photoprotection was vital for the survival of this clone.

**Discussion:**

Despite these differences, ABA metabolism and signaling pathways played a significant role in the drought stress tolerance of both clones. Osmoprotection, osmotic adjustment, and antioxidant response appear to be part of the core strategy of *T. cacao*’s tolerance to water deficit stress. This research provides valuable insights into the distinct molecular mechanisms underlying drought-stress tolerance in cacao plants. Specifically, it identifies stress-tolerance candidate genes of breeding value, as well as for *T. cacao* germplasm characterization, conservation and selection.

## 1 Introduction

Cacao (*Theobroma cacao* L.) is a perennial crop native to the Amazon basin which grows in tropical rainforests across America, Africa, and Asia (Motamayor et al., 2002). Cacao beans are the raw material to produce chocolate, cocoa butter, confections, beverages and cosmetics with high pharmaceutical industry interest (Katz et al., 2011; Scapagnini et al., 2014). Cacao cropping has a significant high and increasing commercial value in global market (Quintero R and Díaz Morales, 2004; Bartley, 2005; Wickramasuriya and Dunwell, 2018), with world production exceeding 5.6 million tons in 2023 (FAO, 2023) and a chocolate industry valued at $100 billion US Dollar in 2021, projected to grow by 4.5% to 5.5% annually until 2027 (Bermudez et al., 2022). Cocoa farming supports over 50 million people worldwide, with approximately 6 million smallholder farmers, relying on plots smaller than 5 ha for their livelihood (Motamayor et al., 2002; Osorio-Guarín et al., 2018; Juby et al., 2021; Bermudez et al., 2022).

*T. cacao* requires humid tropical climates with 1200–2000 mm annual of evenly distributed rainfall (Hernandez et al., 2017; Mensah et al., 2023a). However, increasingly frequent dry seasons often due to global warming reduce productivity and causes economic losses (Medina and Laliberte, 2017; Farrell et al., 2018; Lahive et al., 2019; Hebbar et al., 2020). Soil moisture below 60% fails to meet crop water demand and causes severe drought increasing the risk of permanent wilting when soil water content falls below 25% and soil matric potential reaches -1.5 MPa (Alvim and Alvim, 1977; Moser et al., 2010; Carr and Lockwood, 2011; Mensah et al., 2023b, 2023a). Drought reduces root water potential to -1.35 MPa and leaf water potential (ψ_leaf_) to -1.5 MPa, preventing the plants from meeting its water requirements and inducing water deficit stress (Gilbert and Medina, 2016). Water deficit disrupts mineral nutrient absorption and transport, inhibiting photosynthesis, respiration, and photoassimilate distribution; in addition, it causes stomatal closure which, combined with all the above, reduces the plant growth (Chaves et al., 2009). This stress also increases xylem vessel density, thereby reducing water transport and cell turgor, and disrupting cell division and elongation (Khan et al., 2018). These alterations impair plant development and ultimately affect flowering and grain filling (Almeida and Valle, 2007; Moser et al., 2010; Gateau-Rey et al., 2018).

To combat drought and maintain homeostasis, plants respond through multiple mechanisms, including: reduced stomatal conductance and increased water use efficiency, which minimize water loss; increased antioxidant activity to reduce the accumulation of harmful reactive oxygen species (ROS); osmotic adjustment and increased root biomass, in addition to foliar senescence, helping plants to absorb more water and reduce water intensive foliage (Moradi, 2016; Kaur and Asthir, 2017; Kumar et al., 2018). In Cacao plants, stomatal closure and a higher root/shoot ratio have been described as crucial for conserving water and improving absorption capacity (Santos et al., 2014).

Abscisic acid (ABA) plays a key role in tolerance to dehydration by inducing stomatal closure andactivating transcription factors (e.g., AREB, DREB) that regulate effector genes associated with acclimation mechanisms (Deng et al., 1989; Cabuslay et al., 2002; Kozlowski and Pallardy, 2002; Shinozaki and Yamaguchi-Shinozaki, 2007). The process begins when root cells sense drought stress and release ABA, which then moves through the plant vascular system (Hauser et al., 2017; Kuromori et al., 2018; Takahashi et al., 2020). ABA accumulation triggers secondary messengers and activate TF, leading to the expression of stress responsive genes, including those encoding tonoplast proteins such as aquaporins (Kuromori et al., 2018; Osorio Zambrano et al., 2021). An ABA-independent pathway also exists, in which other TF activate effector genes (Yamaguchi-Shinozaki and Shinozaki, 2006; Shinozaki et al., 2017; Jain et al., 2018). These genes produce proteins with metabolic and structural functions that establish plants’ systemic drought response. Plants lacking these tolerance mechanisms enter permanent wilting and eventually die under severe drought (Shinozaki et al., 2017; Jain et al., 2018).

Unravelling these molecular mechanisms is crucial for identifying candidate tolerance genes as a base for conservation, selection, and breeding programs. This knowledge paves the way for developing climate-resilient crops that can withstand the increasing frequency of drought events caused by irregular rainfall patterns (You et al., 2019; Li et al., 2024).

Transcriptomic approaches like RNA sequencing provide valuable insights into gene expression changes associated with plant responses to environmental stressors such as drought (Dossa et al., 2017). These tools have been widely applied in model plants and major crops to elucidate regulatory networks and stress-responses mechanisms, and to identify genes involved in water stress tolerance (Oono et al., 2014; Zenda et al., 2019). Although numerous studies have reported plant stress responses using physiological or transcriptomic methods, few have combined both. In non-model species such as cacao, the molecular mechanisms underlying drought-stress response remain poorly understood. Despite its economic importance, and the availability of a reference genome (Motamayor et al., 2013; Argout et al., 2017), molecular studies in cacao are limited, with few reports on transcriptomic responses to water deficit (Argout et al., 2008).

To address this gap, we applied an integrated approach combining growth and physiological characterization with transcriptomic (RNA-seq) profiling in three commercial cacao clones with different genetic backgrounds. These hybrid clones were selected based on a previous physiological screening that identified different levels of drought tolerance among clones (Osorio Zambrano et al., 2021).

We hypothesized that, despite the apparent similar tolerance observed for the three selected clones, they would display distinct physiological and molecular response to water deficit. Specifically, we propose that drought resilience in cacao is mediated by clone-specific response mechanisms involving a coordinated regulation of stress-responsive genes, primary and secondary metabolism, and other functional pathways. This integrative framework enabled the identification of candidate genes and functional categories associated with drought tolerance, providing insights into the molecular and physiological mechanisms underlying water stress responses in cacao.

## 2 Materials and methods

### 2.1 Plant material

This study was conducted at Bambusa Station (Geoambiente SAS) in Pacho, Cundinamarca, Colombia (5° 07´ 50´´ north and 74°09´30´´ west, at 1350 m.a.s.l). Water deficit response of three *T. cacao* commercial clones: EET8, ICS60 and TSH565 were evaluated. These three clones were chosen based on a previous screening study involving other commercial clones which identified contrasting physiological responses to DS (Osorio Zambrano et al., 2021). Furthermore, these three clones have different genetic background and belong to the most recommended cocoa clones to be planted and commercialized in Colombia with a 20-30-year projection scope (Cardona, 2010).

Under greenhouse-controlled conditions, five-month-old grafted plants were sown in 10 L black plastic bags filled with silty loam soil (pH 6.5) supplemented with charcoal slag. In accordance with the results of the soil analysis, each plant was fertilized with 5 mL of Agroplus^®^ (Fundases) and 5 g of 15-15-15 (N: P: K) Nutrimon^®^ (Monómeros Colombo Venezolanos, SA) per liter of water. All the plants were watered to field capacity until the water deficit treatment began. During the experiment, environmental data was recorded using a HOBO 8 climatic station (HBOware^®^) installed at 0.3 m from the ground. The daily air temperature ranged from 18 to 33 °C, with an average of 24 °C, while the average relative humidity was 78%. The average vapor pressure deficit (VPD) was 1.1 kPa using the method proposed by Allen et al. (2006) (**Supplementary Figures 1A, B**).

### 2.2 Experimental design and induction of water stress

The treatments were distributed in a split-plot arrangement under a randomized complete block design with three replications; the two water treatments were evaluated in the main plot: the field capacity (well-watered: WW) and drought-stress (DS) treatments. In the subplot, three clones were evaluated: EET8, ICS60 and TSH565 with twelve plants of each clone per subplot. For the WW control treatment, the plants were maintained at 70% of volumetric content of water (VWC) in the soil, measured at 20 cm depth with a TDR-150 (Spectrum technologies^®^). The Ψ_leaf_ was recorded predawn with a Schölander pressure chamber (PMS Model 615, Fresno, CA, United States) and ranged from -0.2 to -0.4 MPa under WW treatment, which is adequate for healthy cacao plants (**Supplementary Figure 2B**) (De Almeida et al., 2016). In the DS treatment, irrigation was suspended for 52 days until the average soil VWC dropped to 25% (**Supplementary Figure 2A**) and the Ψ_leaf_ reached -3.0 to -3.5 MPa, indicative of a severe stress (Almeida et al., 2002; De Almeida et al., 2016) (**Supplementary Figure 2B**). This day was marked as Day 52 (D52) as plants showed visible wilting (maximum stress point). After D52, the plants were rehydrated to a soil VWC of 70% within 24 h. Physiological parameters were recorded seven days later Day 59 (D59) to assess recovery.

For the transcriptomic analysis, on D52, the two most contrasting clones in physiological response to DS were selected. The samples of the third and fourth fully expanded leaves were collected from six plants on EET8 and TSH565 clones under WW and DS treatments, which were immediately frozen in liquid nitrogen and stored in the dark at -80 °C for the subsequent extraction of total RNA.

### 2.3 Physiological parameters evaluated

Measurements were taken on the third or fourth fully expanded leaf of each plant at D52 and D59 following previously established protocols (Osorio Zambrano et al., 2021). Ψ_leaf_ was measured predawn between 05:00 and 06:30 h using a Scholander pressure chamber (PMS Model 615, Fresno, CA, United States), and relative water content in the leaf (RWC) was calculated as RWC (%) = [(FW − DW)/(TW − DW)] × 100 where FW is fresh weight, TW is turgid weight, and DW is dry weight, in six plants per treatment (n = 6). After the onset of stress, net photosynthesis (A), and stomatal conductance (g*_s_*) were measured between 09:00 and 12:00 h using a LI-6400XT Portable Photosynthesis System measurement system (LI-COR Biosciences Inc. NE, United States) in nine plants per treatment (n = 9). Chlorophyll fluorescence (F*_v_*/F*_m_*) was measured on the same leaves where A was recorded (n = 9) previously dark-adapted, between 16:00 and 19:00 h using a Junior-PAM modulated fluorometer (Walz^®^, Effeltrich, Germany) whose parameters were calculated with WinControl-3 software (Heinz Walz GmbH Inc., Effeltrich, Germany). Data of A and g_s_ were used to calculate the efficiency of intrinsic water use (WUE*_i_*).

### 2.4 Growth parameters

At D59 after irrigation resumption of DS plants, nine plants per treatment (n = 9)were harvested to determine dry mass for various plants parts: root weight (DWr), stem weight (DWs), leaf weight (DWl) and total dry weight (DWTo). The same plants used in the gas exchange evaluation were fragmented and dried at 70 °C in an oven (Thermolyne^®^, USA) until a constant dry weight was achieved. All weights were recorded using an analytical balance (Adventurer Ohaus^®^, USA).

### 2.5 Statistical and multivariate analyses

An analysis of variance (ANOVA) was performed to determine the effect of treatments (WW and DS) and clones (EET8, ICS60 and TSH565), on the response of the physiological and growth parameters. An LSD test was subsequently performed to assess significant differences (P ≤ 0.05). Statistical analyses were performed with Statistix 9^®^ software (Analytical software, USA).

A multivariate approach was applied to explore joint variation of transcriptomic and physiological traits. A two-step Principal Component Analysis (PCA) was first conducted on variance- stabilized (VST) transcriptomic data using Deseq2 package in R (Love et al., 2014), and the first five principal components (PCs) (explaining > 83% of the variance), were combined with scaled physiological and growth-related variables for an integrated analysis. Finally, a Multiple Factor Analysis (MFA) was performed integrating transcriptomic and physiological datasets to reveal overall patterns among samples and treatments, using the FactoMineR package in R (Lê et al., 2008).

### 2.6 Transcriptomic analysis:

#### 2.6.1 RNA extraction and transcriptome sequencing

Total RNA was extracted according to previously reported protocols (Chang et al., 1993) with some modifications described previously (Osorio Zambrano et al., 2021). The purity and integrity of the RNA were verified with a Bioanalyzer 2100 (Agilent Technologies^®^). The RNA samples of the three individuals (n = 3) per clone and treatment that presented the highest values of RNA integrity (RIN > 6) were selected for the construction of the cDNA sequencing libraries (**Supplementary Table 8**). A total of 12 cDNA libraries were indexed and generated after polyA RNA enrichment using the NEBNext^®^ Ultra™ kit (New England BioLabs) and were subsequently sequenced with paired ends of 150 bp using a HiSeq platform (Illumina, Inc.).

#### 2.6.2 Bioinformatic analysis

##### 2.6.2.1 Processing of RNA-seq reads and mapping to the reference genome

The quality control (QC) of the reads was carried out according to previously reported flowcharts (Yaya Lancheros et al., 2021) using FastQC and MultiQC tools (Andrews, 2010; Ewels et al., 2016). Cutadapt (Martin, 2011), and SortMeRna (Kopylova et al., 2012) were used to remove low-quality reads (Phred score < Q30) and those with low complexity, adapters, and contaminants (rRNA and bacterial, fungal and human). The trimmed or filtered reads were then aligned to the reference genome of *T. cacao* Criollo Belize B97-61/B2 (Criollo_cocoa_genome_V2, assembly GCA_000208745.2; Argout et al., 2017), using HISAT2 with default parameters. This program was also used in the construction of the reference genome index (Kim et al., 2015). The sequences and annotations of the genome were obtained from the Ensembl Plants database version 45 with the use of the BioMart tool (Kinsella et al., 2011; Howe et al., 2020).

##### 2.6.2.2 Differential expression and over-representation analysis

The gene expression levels for each clone under WW and DS treatment were estimated by counting the number of reads mapped per gene (raw counts) using the FeatureCounts tool (Liao et al., 2014). Subsequent normalization and differential expression analysis was carried out with the DEseq2 package (Love et al., 2014) by calculating, for each gene, the log_2_ fold change (Log_2_FC) of the expression level between the plants under DS and control plants (WW), applying a generalized linear model (GLM) and the Wald statistical test. The Benjamini-Hochberg method was applied subsequently by default in Deseq2 to calculate the false discovery rate (FDR) and adjusted P value (P adj.). Genes with significant differential expression were considered those that presented a value of P adj. ≤ 0.05. For the Over-Representation Analysis (ORA), the genes with both significant differential expression, and Log_2_FC absolute values > 2 were selected.

These differentially expressed genes (DEGs) were subjected to functional ORA via Gene Ontology (GO) and Kyoto Encyclopedia of Genes and Genomes (KEGG) pathway analyses using the AgriGO tool (Tian et al., 2017), iDEP and g:Profiler, respectively (Ge et al., 2018; Raudvere et al., 2019). To extract the enriched GO terms, singular enrichment analysis (SEA) was used, with Fisher’s statistical test (P adj. ≤ 0.05). For the KEGG analysis, the g:SCS algorithm recommended by g:Profiler was used (Raudvere et al., 2019). KEGG pathway and enrichment graphics were generated with SRPLOT (Tang et al., 2023).

#### 2.6.3 Validation of gene expression via qRT–PCR

The expression of DEGs that were identified as tolerance candidate genes based on their molecular and cellular functions, as well as their log_2_FC values and statistical significance, was validated via RT-qPCR. RT-qPCR analysis of the selected genes was performed according to previously reported protocols (Osorio Zambrano et al., 2021) using a Quant Studio 3 Real-Time PCR System (Applied Biosystems, CA, USA). The relative expression levels of the selected genes were normalized with respect to the expression level of the ubiquitin gene via the comparative Ct method (2 ^−ΔΔCt^) (Pfaffl, 2001). The experiment was carried out in five independent biological replicates (individuals) per treatment (n = 5) (**Supplementary Table 6**). The list of primers designed and used in this validation is presented **Supplementary Table 3**. The reference gene used was selected after three-gene stability analysis using RefFinder platform which integrates the ΔCt method, geNorm, NormFinder, and BestKeeper (Xie et al., 2023). Each algorithm generated a stability ranking based on variation among Cq values, and RefFinder calculated the geometric mean of the ranking scores to obtain a comprehensive stability value, which was used to identify the most stable reference gene for normalization and was consistent with results previously obtained in cocoa plants (Gallego et al., 2018) (**Supplementary Figure 6**). Data from the validation of candidate drought-tolerance genes is available in **Supplementary Tables 3**, **6**, **7**, **9**.

## 3 Results

### 3.1 Physiological response to water deficit

#### 3.1.1 Leaf water potential

**Figure 1:**
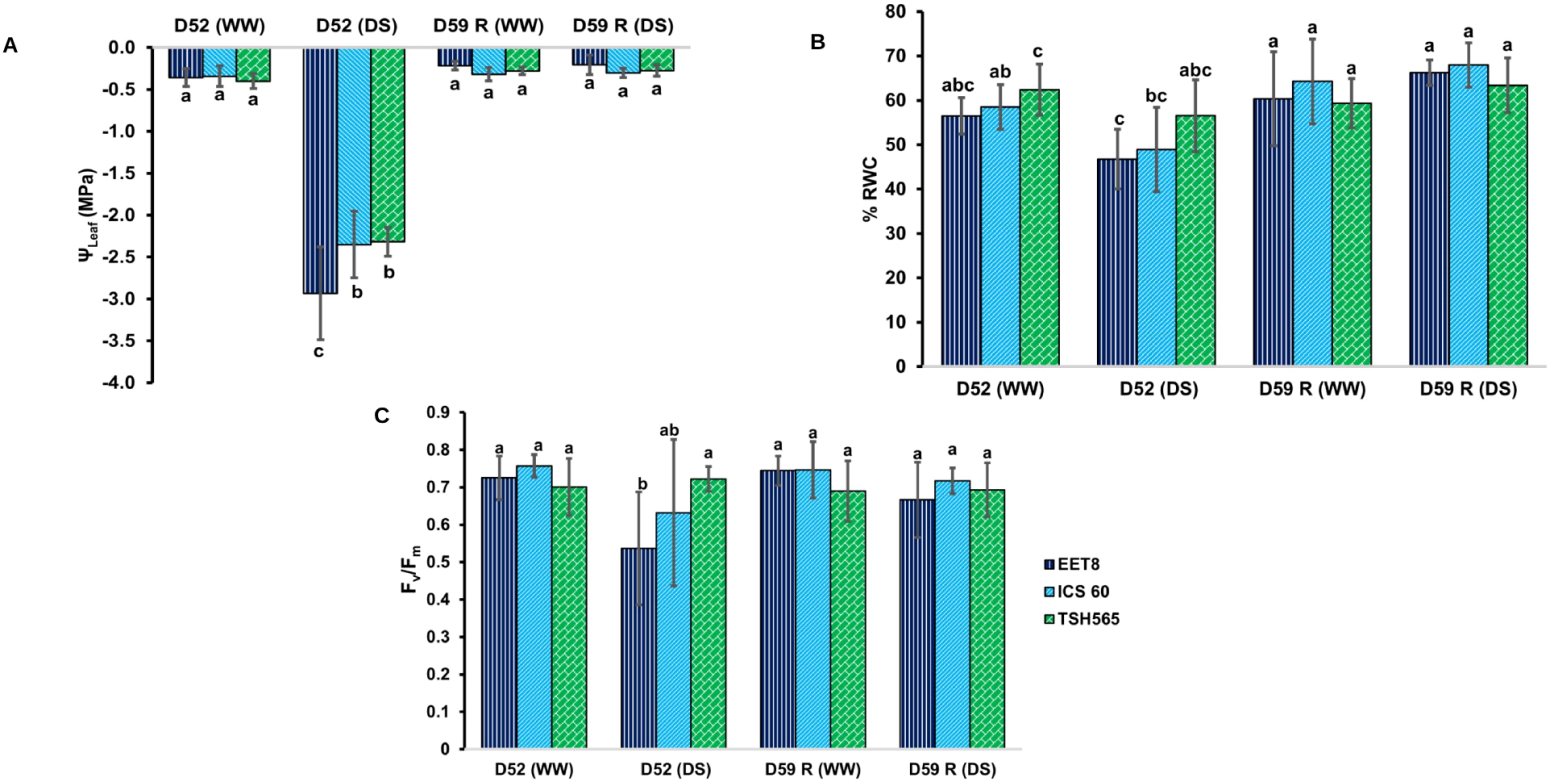
Effect of water deficit (DS) on **(A)** leaf water potential (Ψ_leaf_), **(B)** relative water content in the leaf (%RWC), and **(C)** maximum quantum efficiency of PSII (F*_v_*/F*_m_*), in the *T. cacao* clones EET8, ICS60 and TSH565. Values are means ± SD (n = 6 for Ψ_leaf_ and RWC; n = 9 for F*_v_*/F*_m_*). Significant differences are denoted with different letters produced by two-way ANOVA (clone × treatment). WW: plants irrigated at field capacity; DS: plants subjected to water deficit. D52, day of maximum stress; D59, day on which the measurements were made after 7 days of rehydration.

The Ψ_leaf_ values for the WW group ranged from -0.3 to -0.4 MPa, indicating sufficient hydration. In contrast, under DS, the Ψ_leaf_ dropped to an average of -2.5 MPa. All clones showed significant differences in their Ψ_leaf_ between treatments (P ≤ 0.05). This was accompanied by a significant decrease in soil VWC to 25% (**Supplementary Figure 2A**). At D52, the Ψ_leaf_ between clones varied: TSH565 and ICS60 had significantly greater Ψ_leaf_ (-2.3 MPa) compared to EET8 (-2.93 MPa). After rehydration (D59), the Ψ_leaf_ of the clones under DS recovered to values like those of WW plants indicating that although the three clones experienced a severe water deficit of 52 days, they were able to recover to almost their initial hydric state, which is a trait of drought tolerance (**Figure 1A**).

#### 3.1.2 Relative leaf water content

For WW plants, the RWC was greater (62%) for TSH565 than for EET8 (56%) and ICS60 (58%). At D52, only clones EET8 and ICS60 showed significantly reduced RWC values (P ≤ 0.05) compared to their respective control group (WW): 47% and 49% decrease respectively, whereas TSH565 showed a RWC like unstressed plants. At D59, the EET8 and ICS60 plants subjected to DS reached RWC values like those of their controls, with no differences between clones or between treatments (**Figure 1B**).

#### 3.1.3 Leaf gas exchange and intrinsic water use efficiency

**Figure 2:**
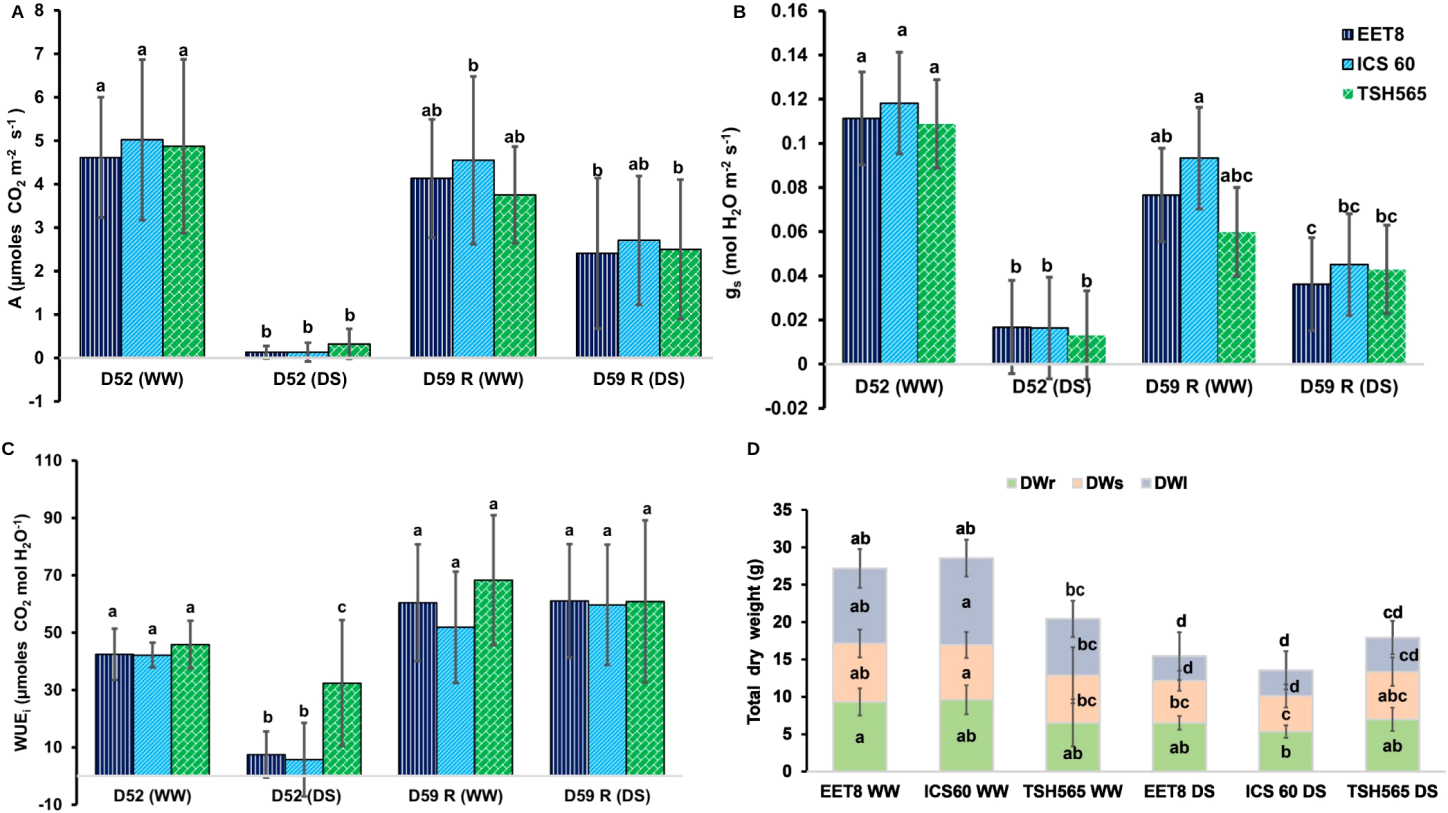
Effect of water deficit (DS) on **(A)** net photosynthesis, **(B)** stomatal conductance **(C)** intrinsic efficient use of water **(D)** dry mass by organs and total mass in the *T. cacao* clones EET8, ICS60 and TSH565. The values are the means ± SD (n = 9). Significant differences are denoted with different letters produced by two-way ANOVA (clone × treatment). WW: plants irrigated at field capacity; DS: plants subjected to water deficit. D52, day of maximum stress; D59, day on which the measurements were made after 7 days of rehydration.

A and *g_s_* were significantly lower (P ≤ 0.05) in DS plants than in the WW plants (**Figures 2A, B**). In WW plants, A was similar between clones: 4.61; 5.0 and 4.87 µmoles of CO_2_ m^-2^s^-1^ for EET8, ICS60 and TSH565, respectively, whereas under DS, A was almost completely inhibited by 97% (0.13 µmol of CO_2_ m^-2^s^-1^) in EET8 and ICS60 clones, while was 91% reduced (0.32 µmol of CO_2_ m^-2^ s^-1^) in TSH565. Interestingly, after rehydration at D59, A recovered around 50% of the values exhibited by their respective control plants in the EET8 and ICS60 clones, while TSH565 exhibited a full recovery, with similar photosynthetic rates to those of its respective controls (**Figure 2A**). On the other hand, the *g_s_* was reduced on average by 85% under DS with respect to the WW plants: dropping in average from 0.113 mol H_2_O m^-2^s^-1^ to an average of 0.015 m^-2^s^-1^. As was observed for A, at D59, EET8 and ICS60 presented *g_s_* values of approximately 50% of the value recorded for the control plants, whereas TSH565 recovered similar *g_s_* values as the WW plants (**Figure 2B**).

Likewise, at D52, the WUE*_i_* was significantly lower (P ≤ 0.05) in the clones subjected to DS than in WW, whose values were 42 µmol of CO_2_ mol H_2_O^-1^ for EET8 and ICS60, and 45 µmol of CO_2_ mol H_2_O^-1^ for TSH565. Under DS, WUE*_i_* was reduced on average by 81% in EET8 and ICS60, whereas TSH565 showed only 29% reduction, with significant differences (P ≤ 0.05) between the clones. At D59, the WUE*_i_* of all clones recovered to the values of the WW plants, without differences between clones or water treatment groups (**Figure 2C**).

#### 3.1.4 Chlorophyll fluorescence

At D52, F*_v_*/F*_m_* decreased to 0.537 (P ≤ 0.05) in EET8 compared with F*_v_*/F*_m_* of WW plants (0.725). A similar reduction pattern was observed for ICS60, with a F*_v_*/F*_m_*dropping from 0.757 (WW) to 0.632 (DS). Although both values indicate photoinhibition, an F_v_/F_m_ close to 0.5 (EET8) reflects a more critical physiological state than 0.6 (ICS60). Interestingly, in TSH565, F*_v_*/F*_m_* was not significantly reduced (LSD, P > 0.05); between WW and DS groups. Similarly, at D59, the F*_v_*/F*_m_* of EET8 and ICS60 clones recovered in average 92% of the baseline value of WW, whereas TSH565, recovered the reference values of the control plants (**Figure 1C**).

#### 3.1.5 Growth parameters

Under DS, the accumulation of DWt was significantly reduced for clones EET8 and ICS60, with values 43% and 53% lower, respectively, compared to WW plants. This decrease was approximately 12% for clone TSH565 and was not statistically significant. For EET8 and ICS60 plants subjected to DS, the DWr, DWs and DWl were all reduced, indicating affectation of growth in all their organs, whereas for TSH565, only DWl was reduced, passing from 7.55 g to 4.57 g (39.5% reduction). Comparatively, for EET8, this decrease was 67%, ranging from 10.08g (WW) to 3.28g (DS), while ICS60 showed 70% reduction, ranging from 11.64g (WW) to 3.42g (DS). For these clones, the DWr, also decreased 30% and 40% respectively under DS, exhibiting a similar reduction of their DWs (28% and 35%, respectively) compared to WW plants. In contrast with TSH565, DWr and DWs did not differ between treatments (**Figure 2D**).

### 3.2 Transcriptomic analysis of *T. cacao* under DS

Although ICS60 and EET8 showed statistically similar responses for several physiological parameters, their absolute F_v_/F_m_ values revealed different levels of photochemical impairment under DS. TSH565 maintained F_v_/F_m_ values closer to those expected for non-stressed plants, indicating minimal photochemical damage, ICS60 showed intermediate F_v_/F_m_ values, whereas EET8 exhibited the lowest Fv/Fm values, approaching the critical range associated with severe photosystem impairment. Regarding their genetic backgrounds, TSH565 (a Trinitarian hybrid) and EET8 (an Ecuadorian “Criollo” hybrid) are also more genetically divergent than any of them with ICS60, which is a Trinitarian x “Criollo” hybrid. Therefore, transcriptomic profiling carried out on the most genotypically and phenotypically contrasting clones: TSH565 and EET8, to capture the largest physiologically meaningful contrast in cacao drought response.

Paired-end sequencing of the 12 RNA-seq libraries generated a total of 354 million raw read pairs (106.2 Gb). After quality trimming, the same number of read pairs were preserved, as only a very small fraction of low-quality bases and residual adapter sequences were removed from the reads ends due to the overall high quality of the sequencing data (Q = 37). On average, each library contained 29.5 million clean reads pairs. The sequences were aligned to the Criollo Belize V2 reference genome with a mapping percentage of 92% on average, resulting in an average coverage per gene of 120X. Similarly, on average, 88% of the readings from each library were assigned to one of the 24561 genes present in the EnsemblPlants database of the reference genome used (Kinsella et al., 2011; Howe et al., 2020) (**Supplementary Table 4**).

Quality assessment of transcriptomic data included PCA and hierarchical clustering, which showed clear separation by treatment and genotype, with enhanced genotype discrimination under drought stress versus control conditions, revealing distinct stress-responsive transcriptomic profiles. Diagnostic evaluation of expression data via MA plot confirmed proper normalization without technical biases, while Volcano plot analysis showed appropriate distribution of DEGs without anomalous patterns in significance or expression fold-changes, validating the dataset for differential expression analysis and biological interpretation (**Supplementary Figure 7**).

**Figure 3:**
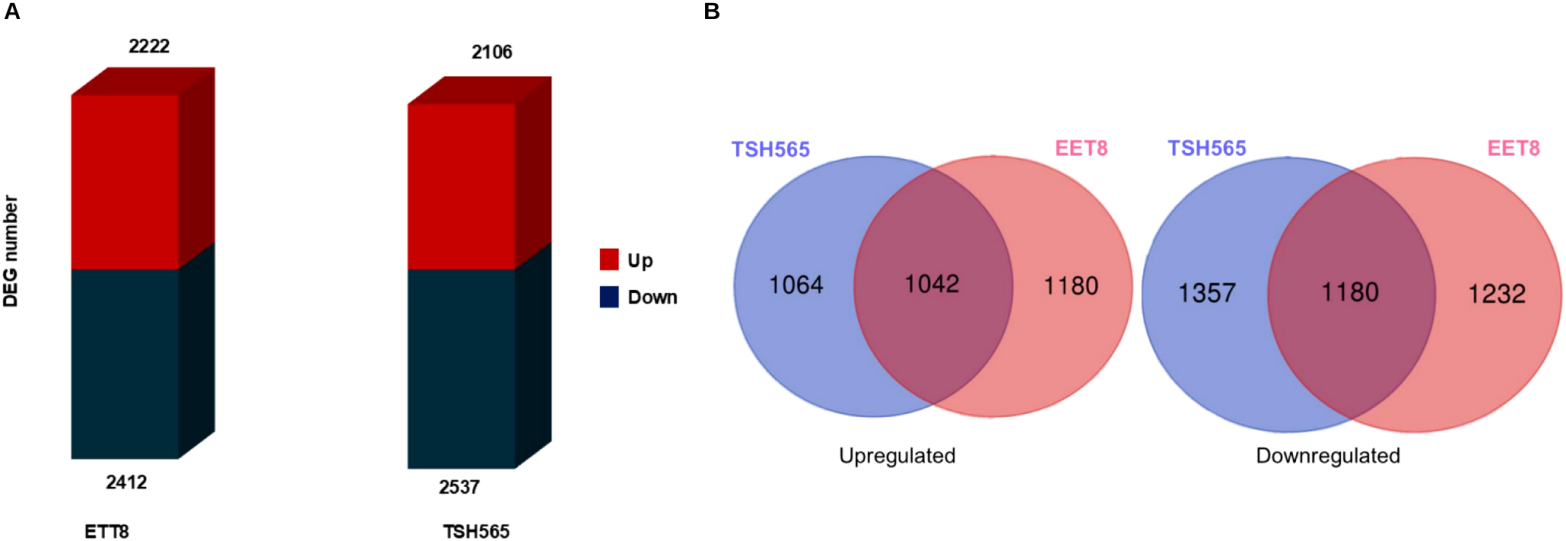
Differential gene expression analysis of *T. cacao* TSH565 and EET8 clones subjected to 52 days of water deficit. **(A)** Bar graph of all the DEGs (P adj. ≤ 0.05) upregulated (red) and downregulated (blue) in the two clones under DS (compared to WW condition) **(B)** Venn diagrams show the number of shared and unique DEGs upregulated and downregulated under DS.

DE analysis (DS vs. WW plants) was performed for TSH565 and EET8 clones and log_2_FC was calculated for each gene. DEGs were filtered according to their significance (P adj. ≤ 0.05). A similar number of DEGs was found for both clones, with slightly fewer DEGs for EET8 (4634) than for TSH565 (4643). Among the DEGs, 2222 and 2106 genes were upregulated, whereas 2412 and 2537 genes were downregulated in EET8 and TSH565, respectively (**Figure 3A**). Common DEGs were identified between the clones, and others were specific to each clone. Among the genes shared, 1042 were upregulated and 1180 downregulated, whereas for the DEGs exclusive to TSH565, 1064 were upregulated and 1357 downregulated, while for EET8, 1180 were upregulated and 1232 downregulated (**Figure 3B**).

**Figure 4:**
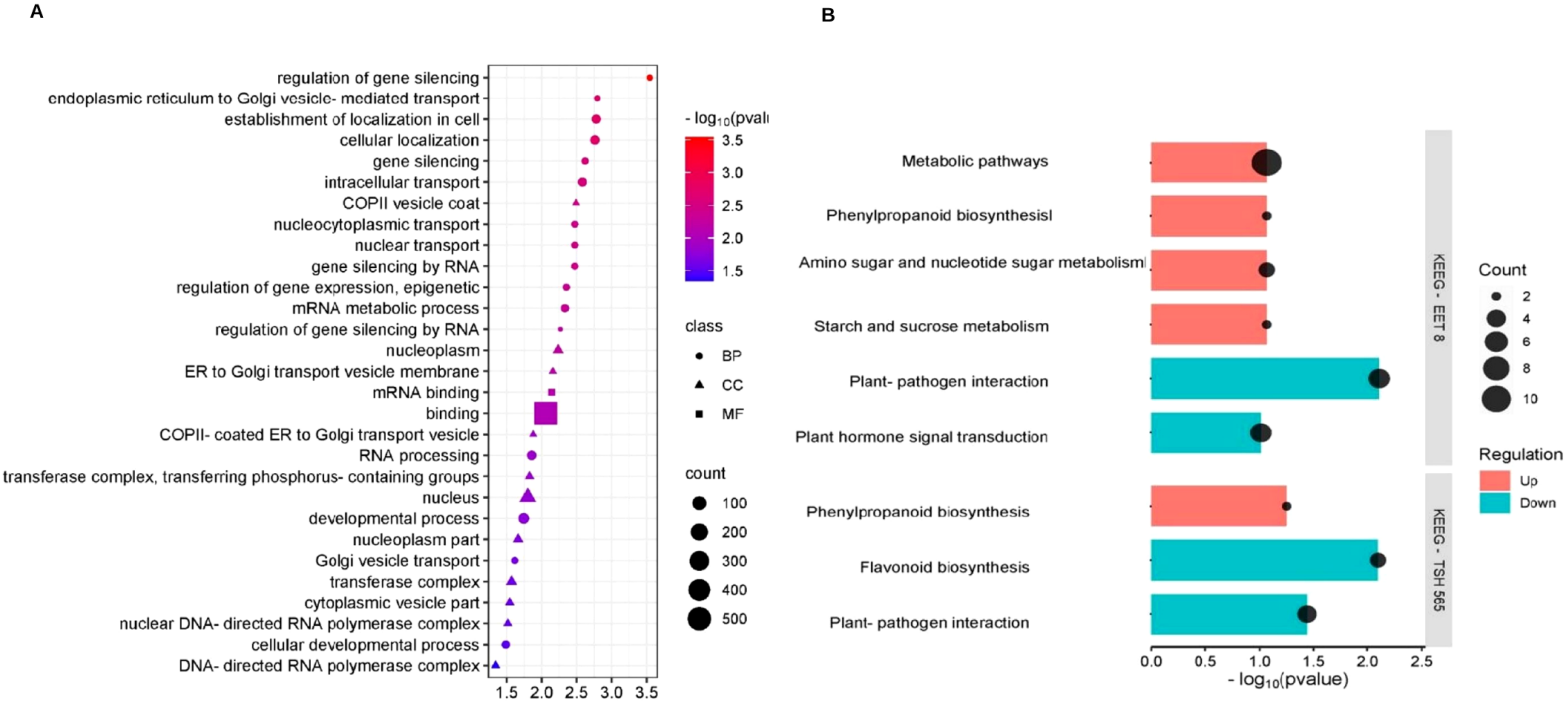
Over representation analysis of GO terms and KEGG pathways in the response of *T. cacao* to water deficit. **(A)** GO terms enriched in the upregulated DEGs under DS (P adj. ≤ 0.05) common to the clones TSH565 and EET8. **(B)** KEGG pathways enriched in the upregulated and downregulated genes (log_2_FC ≥ 2) under DS in the two clones evaluated.

Next, to functionally annotate the common DEGs between clones, an Over Representation Analysis (ORA) based on both GO terms was performed (**Figure 4A**). The GO biological process terms enriched in the 1042 DS-upregulated DEGs, common to both clones, were associated with stress response and regulation of gene expression at different levels: epigenetic, regulation of gene silencing, mRNA metabolic processes, and RNA processing. In addition, intracellular transport, such as transport to and from the nucleus, as well as transport between the endoplasmic reticulum (ER) and the Golgi apparatus mediated by vesicles (COPII), together with cellular development processes were enriched. The main enriched molecular function term was binding to mRNA and, in general, binding to other molecules, and the main enriched cellular compartments terms were the nucleus, vesicles (COPII) that transport substances from the ER to the Golgi apparatus, RNA polymerase complexes, and transferase complexes (**Figure 4A**).

#### 3.2.1 Identification of candidate genes involved in tolerance to DS

To identify differences in the responses of each clone to DS, the DEGs were further explored by applying a minimum threshold on the absolute value of the log_2_FC > 2. With this filter, there were 328 upregulated genes in TSH565 and 404 genes in EET8. Among this set of genes, 136 were common to both clones, whereas 192 and 268 genes were unique to TSH565 and EET8, respectively. The total number of genes downregulated in clone TSH565 was 699, and 782 in EET8. Among these, 332 genes were common to both clones, whereas 367 were unique to clone TSH565 and 450 were unique to clone EET8.

The upregulated and downregulated genes in response to DS were analyzed via KEGG pathway ORA analysis: interestingly, the phenylpropanoid biosynthesis pathway was upregulated in both clones (**Figure 4B**). Additionally, in the EET8 clone, different metabolic pathways, the metabolism of amino acids and nucleotides, and the metabolism of starch and sucrose were also well represented among up-regulated genes. With respect to the DS -downregulated genes, the plant–pathogen interaction pathway was represented among the common downregulated DEGs. More specifically, the plant hormone signal transduction pathway was downregulated in EET8, whereas in TSH565, the biosynthesis of flavonoids was significantly represented among the downregulated genes (**Figure 4B**).

Using the filtered genes, the GO analysis of the common upregulated genes revealed that the biological process terms response to stress, response to stimuli, defense response, response to levels of oxygen, and biosynthesis of flavonoids were the most represented. The enriched molecular function terms were binding to other molecules and catalytic functions. Overall, the genes with the greatest change in expression, which are shared by both clones, were related to the stress response (**Supplementary Figure 3A**).

Regarding downregulated biological processes common to both clones, GO ORA analysis revealed genes related to terms like cell morphogenesis, cell wall biogenesis, response to osmotic stress and abiotic stimulus and response to salt stress; the enriched cellular component term was the extracellular region (**Supplementary Figure 3B**). In accordance with these findings, DS caused downregulation of genes associated with growth processes, as well as a response to other types of stress, suggesting that DS negatively affects these processes similarly in both clones.

**Figure 5:**
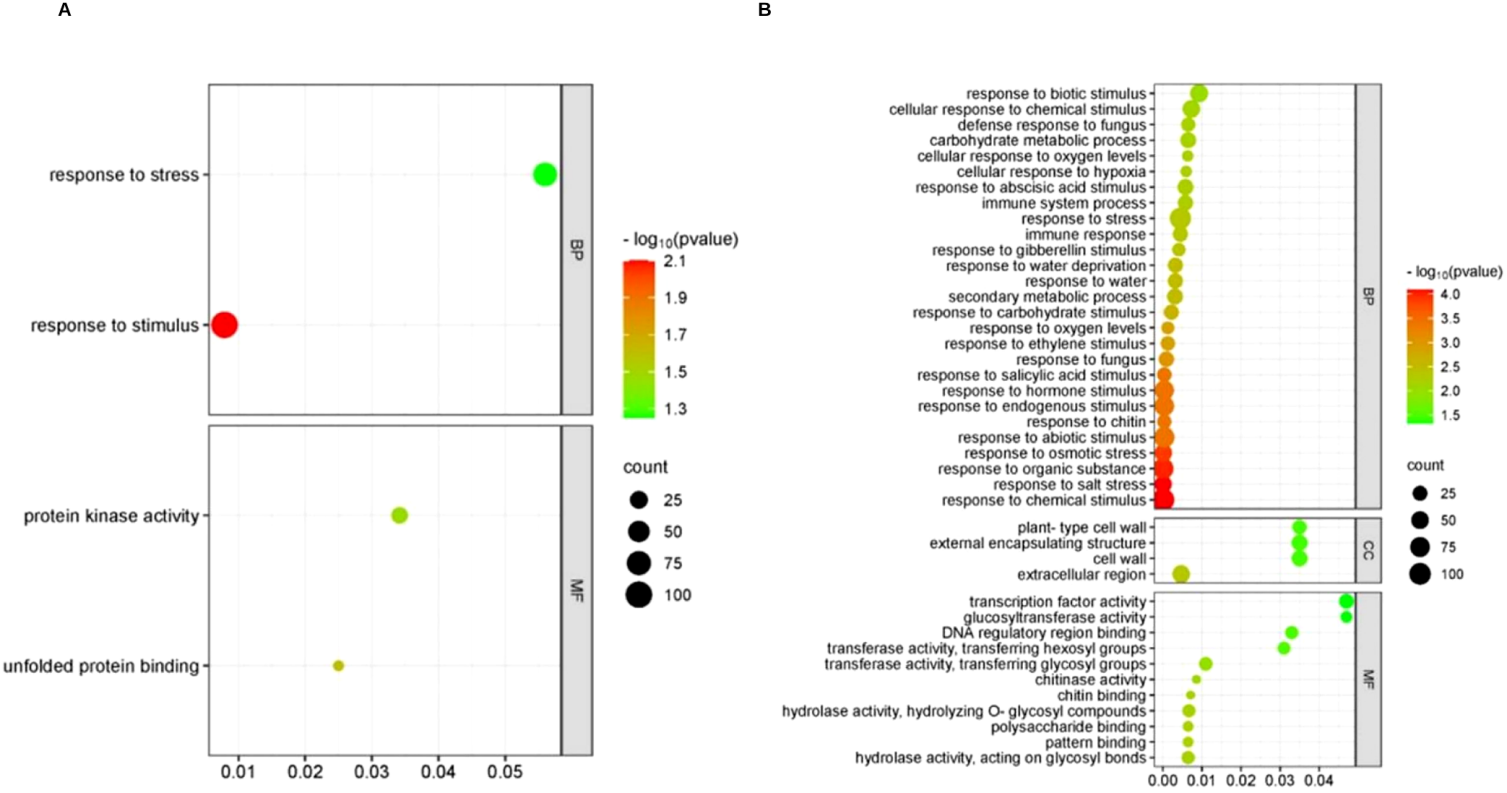
GO terms enriched in the genes associated with response to water deficit in each clone. **(A)** GO terms enriched in upregulated genes (log_2_FC ≥ 2) in response to DS in clone TSH565. **(B)** GO terms enriched in genes with upregulated (log_2_FC ≥ 2) in response to DS in the EET8 clone.

The GO analysis of the 192 upregulated genes in TSH565 revealed only two overrepresented biological process terms: response to stimuli and response to stress. The enriched molecular function terms were related to binding unfolded proteins and the activity of protein kinases (**Figure 5A**). Several genes within these categories are annotated as components of ABA-related signaling pathways.

The GO analysis of the downregulated genes in TSH565 revealed that the biological process terms flavonoid metabolism, shoot development, response to jasmonic acid, leaf morphogenesis, and responses to external stimuli, among others, were overrepresented (**Supplementary Figure 4A**).

On the other hand, in EET8 clone, ORA of GO terms revealed up-regulation of genes related to biological process like response to chemical stimulus, response to osmotic stress, response to hydrogen peroxide, response to lack of water, response to saline stress, response to abiotic stimulus, response to stress, response to stimulation by ABA, defense response, response to growth regulators such as ethylene and gibberellins, and secondary metabolic processes, among others (**Figure 5B**). The enriched molecular function terms associated with these processes included binding to other molecules, catalytic functions, and the activity of transcription factors. Importantly, same genes could be involved in various processes, such as stress response, response to stimulation by ABA, and response to lack of water and abiotic stress. For instance, several transcription factors were found among the upregulated DEGs for this clone: NAC, WRKY, MYB, ERF/AP2, SRG1, CRF2, bHLH, Zinc finger_C2H2, ARF5, IAA6, and ERF114, which are all associated with the response to water deficit in plants and the ABA-dependent and ABA-independent pathways.

The GO analysis of the downregulated genes in this clone revealed that the biological process terms regulation of hormone levels, transport of hormones, response to auxins, ethylene, and stimuli, endogenous developmental processes, tropism and gravitropism, and cell wall biogenesis were overrepresented (**Supplementary Figure 4B**).

Finally, considering the high number of DEGs identified and the great diversity of biological processes involved in the response to DS, either in common or unique to each clone, we performed a non-exhaustive selection of the most functionally relevant DEGs associated with these GO categories and KEGG pathways, which supported the response of tolerance to DS in *T. cacao* (**Supplementary Tables 1**, **5**). We corroborate these functions through orthologous relationships with genes from other model plant species, and the literature, to propose them as drought-stress tolerance candidate genes in cacao.

Thus, the genes with the greatest log_2_FC under DS were related to osmoprotection, cell growth, synthesis of the ABA precursor, transport, signaling, defense, transcription factors, and protection from oxidative damage via the antioxidant system. Furthermore, a more directed search was conducted on genes related to the antioxidant system, osmolyte biosynthesis and PSII function, based on both the results of this research and previous physiological characterizations of drought response in cacao plants (**Supplementary Table 2**).

Additionally, the differential expression of the genes reported in the literature, as either target genes or interacting partners of those selected based on their relatively high log_2_FC values (**Supplementary Tables 1**, **2**) was verified. Among the DEGs identified as most relevant (based on significance, Log_2_FC, or literature support), eight genes were selected to validate the RNA-seq expression profile using RT-qPCR (**Supplementary Figure 5A**). The RT–qPCR results were consistent with the RNA-seq data, except for one gene (FER, **Supplementary Figure 5C**). Changes in relative expression showed a positive correlation (r = 0.63; P = 0.01) between the two methods (**Supplementary Figure 5B**), thereby validating the workflow and analytical parameters applied in the DE analysis.

## 4 Discussion

### 4.1 Response of *T. cacao* to water deficit

Cacao plants respond to water deficit through a complex network that integrates biochemical and molecular processes with an efficient signal transduction system, at both subcellular and systemic scales. This study integrated transcriptomic (RNA-seq), growth and physiological analysis to better understand this complex response, providing valuable insights and contributing to filling a knowledge gap in the cacao tolerance mechanisms to drought, an agronomically important trait that has not been well studied in this crop. Stress due to water deficit was successfully induced in the clones as evidenced by the reached values of VWC, RWC and Ψ_leaf_, values similar to those found in cacao plants growing in field (agroecosystems) under irregular rainfall patterns or prolonged dry season and reported to negatively impact crop productivity (Gateau-Rey et al., 2018; Jaimez et al., 2021). The drought tolerance of the clones studied had been confirmed previously (Osorio Zambrano et al., 2021).

As expected, suspending irrigation for 52 days reduced the VWC to 25%, proposed as the permanent wilting point for cacao plants, corresponding to a soil matric potential of -1.5 MPa (Moser et al., 2010). Below this threshold, capillary action in the roots is inhibited, water uptake ceases, and the plant cannot recover after rehydration (Moser et al., 2010). Under DS treatment, the Ψ_leaf_ decreased to -2.93 MPa in EET8 and -2.3 MPa in TSH565 and ICS60, evidencing a more pronounced stress in EET8 (**Figure 1A**). Ψ_leaf_ is an indicator of water status and cellular turgor, which are essential for photosynthesis, survival and growth of plants and are directly related to stomatal closure; here, the g_s_ reduced to 0.015 mol H_2_O m^-2^s^-1^ on average for all clones (**Figure 2B**). This stomatal closure is associated with the tolerance of cacao to drought, as a well-known drought avoidance mechanism in vascular plants that reduces water loss and delays wilting, but at the expense of CO_2_ assimilation and photosynthesis due to stomatal limitation (Almeida and Valle, 2007; Osakabe et al., 2014; Shinozaki et al., 2017; Haghpanah et al., 2024).

Consistent with this response, A declined to 0.13 µmol of CO_2_ m^-2^s ^-1^ in EET8 and ICS60, while in THS565, A was 0.32 µmol of CO_2_ m^-2^s^-1^ corresponding to reductions of 97% and 91%, respectively. Notably, TSH565 maintained higher RWC (62%, like WW plants), indicating superior water retention capacity and physiological resilience under DS. These findings highlight TSH565 as a more tolerant clone to water deficit.

In EET8 and ICS60, A declined progressively with prolonged DS, accompanied by a nonstomatal limitation associated with the inhibition of the photosynthetic apparatus, as evidenced by a significant reduction in F_v_/F_m_, whereas TSH565 showed minimal change (**Figure 1C**). Notably, the strongest physiological contrast was observed between EET8 and TSH565, highlighting differential levels of photoinhibitory damage under DS. In this context, reductions of F_v_/F_m_ to 0.5 are considered physiologically more critical than values around 0.6, as they reflect non-linear declines in PSII efficiency associated with structural damage and inactivation of reaction centers rather than reversible regulatory limitations (Maxwell and Johnson, 2000; Baker, 2008). The higher tolerance of TSH565 to DS is also corroborated by its ability to maintain higher WUE*_i_*, which was reduced by only 29% compared with an 84% in EET8 and ICS60, reflecting a greater ability to sustain photosynthesis under water limitation (Oguz et al., 2022) (**Figure 2C**). Because WUE*_i_* integrates the balance between carbon assimilation and stomatal regulation, differences in their ratio may arise even when variation in A or g*_s_* does not reach statistical significance (Gilbert et al., 2011; Singh and Reddy, 2011).

This physiological response is regulated by ABA, which modulates stomatal closure and transpiration and consequently WUE*_i_*. Reduced A and WUE*_i_*, in EET8 and ICS60 led to lower photoassimilate translocation and growth, significantly decreasing DWTo. In contrast, TSH565 plants under DS showed dry weight reduction only in leaves (DWl), response related with smaller leaf size to limit transpiration, while maintaining root growth for water uptake increase, conferring greater tolerance to drought (Oguz et al., 2022) (**Figure 2D**). This coordinated response is consistent with the downregulation of genes related to shoot development, leaf morphogenesis, and biotic stress observed in DEG analysis. In this context, because the cacao plants analyzed here were grafted, clone comparisons were restricted to scion responses, and transcriptomic analyses were therefore conducted in leaf tissues, which directly reflect the physiological traits evaluated. Root transcriptomic response was not characterized here, as the rootstock for all evaluated clones was genotypically identical and would not have served the main objective of our research: finding specific differences in response to drought between different genotypes of *T. cacao*. Yet, extending these approaches to root tissues in experimental systems specifically designed to assess rootstock-scion interactions and their contribution to DS tolerance represents a relevant direction for future research.

Growth of all organs in the EET8 clone markedly decreased under DS, which was accompanied by the downregulation of genes associated with growth and development processes, as well as pathways related to tropism and gravitropism. This transcriptional pattern was consistent with the reduced growth parameters found for this clone.

**Figure 6:**
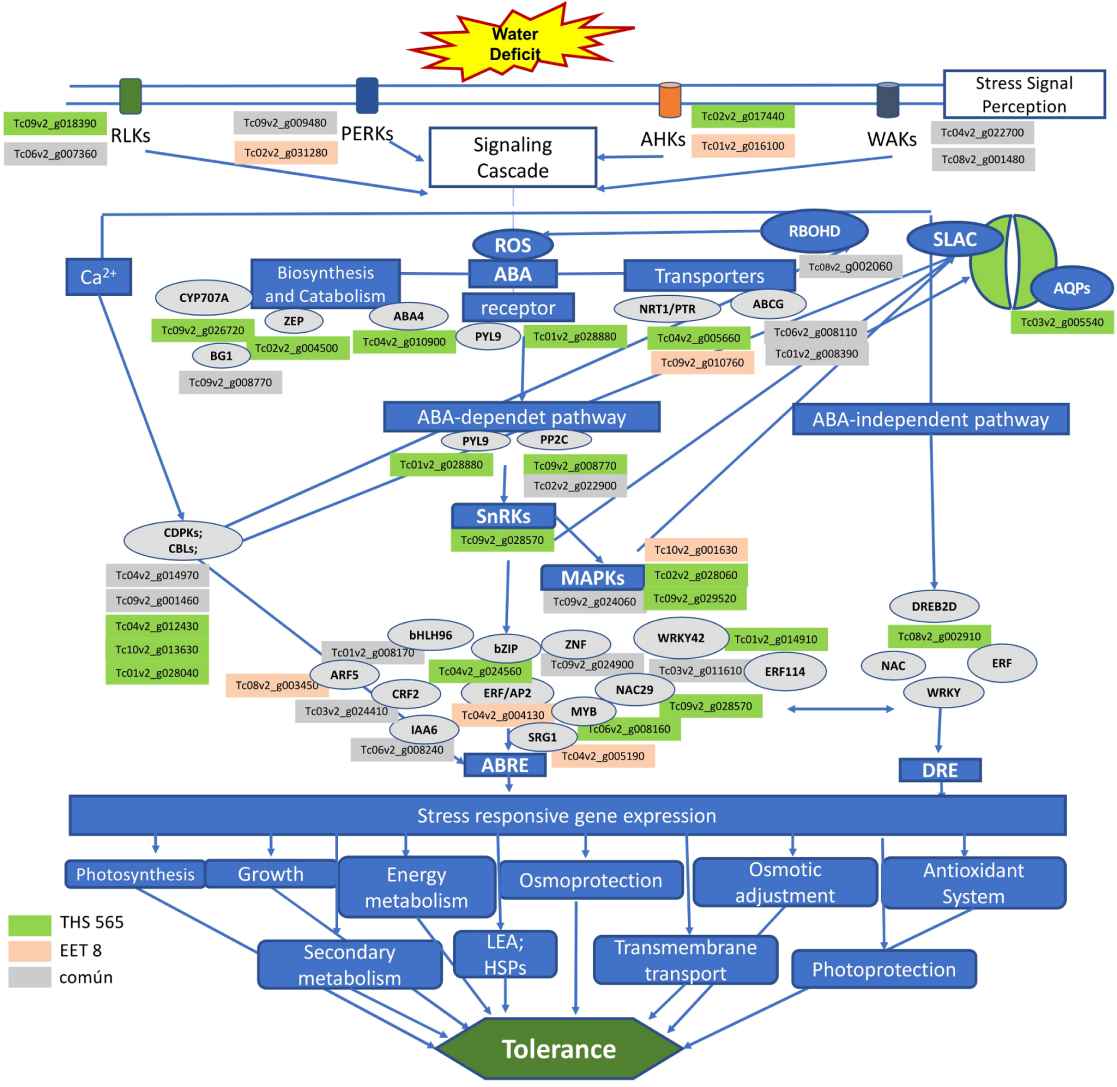
Signaling cascade and activation of effector genes in *T. cacao* in response to water deficit. The green boxes contain the genID of the genes induced in clone TSH565 in response to DS, the red boxes correspond to the genes induced in EET8, and the gray boxes correspond to common genes induced in the two clones. The gray ovals represent the genes involved in each stage of the signaling cascade. At the bottom, in the blue boxes, the mechanisms and processes activated by the genes expressed under DS that contribute to the tolerance of *T. cacao* to water deficit are mentioned.

All clones exhibited recovery after one week rehydration (D59) (**Figures 1**, **2**). These indicate drought tolerance supported by ABA signaling, damage repair at the cellular level, antioxidant system activity, and the accumulation of osmoprotective compounds and other biosynthesized proteins and molecular chaperones, which, together, allow the plant to overcome DS conditions (**Figure 6**) (Deng et al., 1989; Chen et al., 2016; Dinesh et al., 2016; Liao et al., 2017).

The physiological and morphological changes exhibited by the cacao clones during prolonged drought involved several tolerance mechanisms, as evidenced by the changes in gene expression patterns revealed through transcriptomic analyses. These analyses provided high resolution insights into the differential tolerance responses between clones, despite belonging to the same species and being exposed simultaneously to DS under the same growth conditions. Although DE analysis revealed that common mechanisms as evidenced by shared DEGs, the substantial number of unique DEGs in each clone suggests that different genetic programs can lead to a similar outcome: tolerance to severe, prolonged DS followed by full recovery within days of rehydration (**Figure 6**).

**Figure 7:**
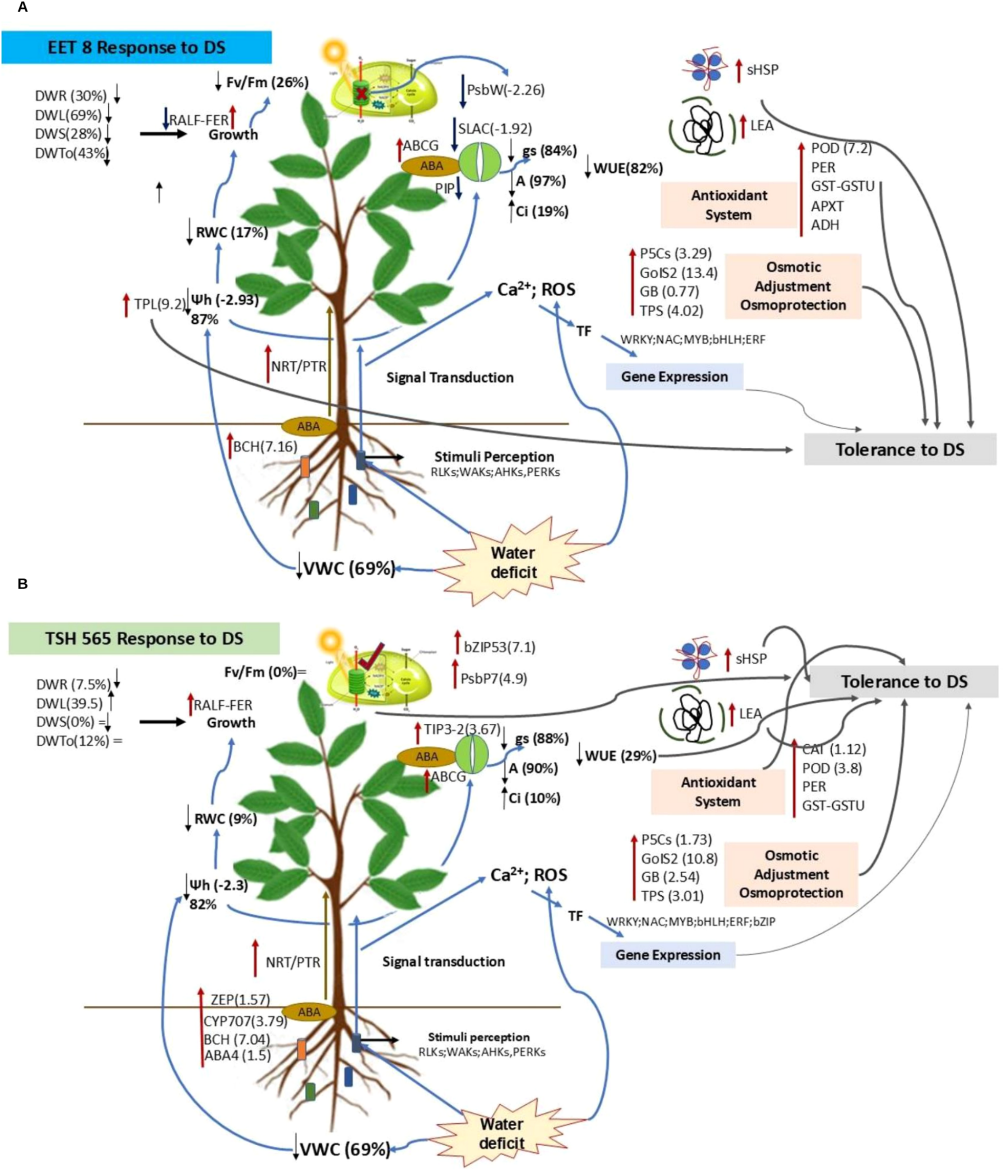
Integrated and comparative model of the response of *T. cacao* to water deficit. **(A)** Response of EET8 clone to DS. **(B)**. Response of TSH565 clone to DS. The red arrows refer to the genes whose expression was upregulated in response to DS, the dark blue arrows represent the downregulated genes, and the directions of the black arrows (upward and downward) correspond to the direction of expression changes due to DS. The symbol (=) refers to the fact that there was no change in that parameter by the DS. The light blue smoothed lines show the direction of the response starting at the root. The gray lines highlight the main genes and mechanisms that contribute to the response to DS in each clone.

In TSH565, the reduction in A under DS was due solely to stomatal limitation. However, the DEGs in this clone triggered multiple responses including stomatal closure and reduced leaf size to minimize water loss by transpiration, enhanced root development to improve water uptake, and increased biosynthesis of metabolites and antioxidant compounds to protect against dehydration. These coordinated strategies deployed by this clone to respond to DS were triggered to keep the photosynthetic machinery functional and an efficient use of limited water (**Figure 7B**).

At the molecular level, several core mechanisms involved in the response of *T. cacao* to DS are conserved with those described in model species, mainly those associated with stress recognition and signal transduction, as well as the activation of common effector genes. However, expression profiles vary with genotype, developmental stage, severity and duration of stress (Farooq et al., 2009; Zenda et al., 2019). Consequently, the identification of DEGs unique to each evaluated clone, along with distinct enriched GO terms and KEGG pathways, was critical for elucidating the main differences underlying their contrasting tolerance responses (**Figure 6**).

### 4.2 Genes involved in the water deficit tolerance of *T. cacao*

Variation in the expression of common and clone-specific genes in response to DS was related to different biological processes and functions involved in key stages of stress signaling cascades and gene activation. Upon sensing the stress signal (soil matric potential ≤ -1.5 kPa), osmosensors and hydraulic sensors in root cell plasma membranes detect the reduced osmotic and water potential caused by soil VWC (< 15%). This signal is then transduced, generating secondary messengers (ROS, Ca^2+^, cyclic nucleotides, inositol phosphates) that trigger a phosphorylation and dephosphorylation cascades that activate transcription factors responsible for expressing stress-response genes, whose products drive physiological and biochemical mechanisms that confer tolerance or acclimation (Shinozaki et al., 2017; Jain et al., 2018).

Abscisic acid (ABA) also plays a central role in this response. Under water deficit, ABA accumulates in vascular tissues and is rapidly mobilized via apoplastic pathway (Mo et al., 2024). This phytoregulator triggers a two-phase response: a rapid stomatal closure to minimize water loss, followed by a slower ABA-dependent signaling cascade that activates long-term adaptive mechanisms to sustain drought tolerance (Geiger et al., 2011) (**Figure 6**).

#### 4.2.1 Signal recognition and transduction

Protein kinases and phosphatases play a fundamental role in stress recognition and signal transduction. In both clones, several of these genes were found to be upregulated and downregulated (**Figure 6**; **Supplementary Table 1**), including receptor-like kinases (RLKs) involved in hydraulic signals perception, wall-associated protein kinases (WAKs), linked to cell wall extension; proline-rich extension-like kinases (PERKs), essential for maintaining cell wall integrity, and histidine kinases (AHKs), responsible for sensing turgor changes (Christmann et al., 2013; Shinozaki et al., 2017). The overexpression of these receptors highlights their role in mediating the drought response in *T. cacao* and underscores the complexity of signaling machinery deployed by these clones to perceive and trigger a response to DS. Conversely, the downregulation of some receptor genes aligns with previous reports describing the complex and dynamic regulation -both positive and negative- within the signaling networks that orchestrate drought stress responses (Zenda et al., 2019).

Among the genes that are most strongly induced by DS in both clones was a histidine phosphotransferase (HP; Tc09v2_g024060) (**Supplementary Tables 1**, **5**). HPs are key elements of two-component signaling systems, transferring phosphorylation signals of histidine kinases (HKs) to response regulators. This DS-induced gene is an ortholog of the rice gene OsAHP1, a positive regulator of cytokinin-mediated signaling pathways that contribute to drought and salinity tolerance and promote root elongation (Sun et al., 2014; Liu et al., 2017).

Similarly, in both clones, two genes encoding FERONIA receptor kinases (Tc10v2_g001630 in EET8 and Tc02v2_g028060 in TSH565) were highly upregulated (**Supplementary Table 1**). FER plays a critical role in vegetative growth regulation through its interaction with the rapid alkalinization factor (RALF) peptide (**Supplementary Table 2**) (Wang et al., 2020a). It also acts as a signaling hub integrating pathways related to cell expansion, energy metabolism and stress response, making it a versatile regulator of growth and survival (Liao et al., 2017). A mechanism involving FER activation by ABA and interaction with RALF has been proposed to coordinate growth and stress signaling (Chen et al., 2016).

Interestingly, while FER was upregulated in EET8 and TSH565 clones, RALF expression diverged: it was upregulated in EET8 (Tc01v2_g024780) but downregulated in TSH565 (Tc05v2_g025930). This differential regulation suggests that the FER-RALF interaction in EET8 may have contributed to reduced root growth under DS, whereas in TSH565, FER induction combined with RALF repression supported continued root development (a 7.5% increase in DWr), enhancing water uptake and maintaining cellular turgor. Given FER´s multifunctional role in stress adaptation, it emerges as a strong candidate gene for mediating drought tolerance in *T. cacao* (**Figure 7A**).

In the MAPK signaling pathway, MAPK protein cascades are sequentially activated through the phosphorylation of at least three mitogen-activated protein kinases (MAPKKK, MAPKK, and MAPK). This pathway is a key regulator of plant development and response to stress (Dinesh et al., 2016). Here, the gene encoding MAPKKK13(Tc09v2_g029520), involved in the first step of this cascade, was highly expressed in the TSH565 clone (**Supplementary Table 1**). This gene is orthologous to *Arabidopsis thaliana* MAPKKK14, a Kinase implicated in ABA-mediated signaling pathway (Sah et al., 2016). Consistent with findings in other species the upregulation of MAPK cascade genes in response to stress highlights their central role in adaptative responses and underscores their potential as targets for improving drought tolerance in crops (Wang et al., 2015; Jia et al., 2016; Ye et al., 2017; Muhammad et al., 2019).

#### 4.2.2 The role of ABA in the response of *T. cacao* to water deficit

This study highlights the crucial role of abscisic acid (ABA) in the drought stress response of two T. cacao clones to DS, EET8 and TSH565. Both clones activated several ABA-related genes to cope with water deficit (**Supplementary Tables 1** and **2**). A key finding was the upregulated of beta-carotene 3-hydroxylase1 (BCH, chloroplastic), an enzyme essential for synthesizing zeaxanthin, which is a precursor to ABA. Zeaxanthin is a vital component of the xanthophyll cycle, a mechanism that helps protect thylakoid membranes and dissipates excess light energy as heat (Du et al., 2010). Studies in rice and Arabidopsis have shown that manipulating the BCH gene can improve drought tolerance by boosting xanthophyll cycle activity and ABA-mediated responses, suggesting a similar function in *T. cacao* (Davison et al., 2002).

Additionally, the gen Tc05v2_g012420, encoding glucan endo-1,3-beta-glucosidase (an ortholog of the Arabidopsis BG1 and BG2), was strongly upregulated in both clones, with higher expression in EET8 (7.5-fold) (Shinozaki et al., 2017). This enzyme likely hydrolyzes ABA-glycosyl ester (ABA-GE), a storage form of ABA, releasing active ABA during DS, contributing to stomatal closure for improved water conservation.

In TSH565 clone exhibited a more robust response, activating both ABA-dependent and independent pathways. It showed increased expression of genes involved in ABA synthesis and catabolism, including ZEP (zeaxanthin epoxidase), CYP707A2 (abscisic acid 8′-hydroxylase 2), and ABA4 (ABA DEFICIENT 4, chloroplastic), along with the extracellular ABA receptor PYL9 (Tc01v2_g028880). It also upregulates DREB2D, a transcription factor linked to ABA-independent DS in melon (Ansari et al., 2017) and clones of *Camellia sinensis*, a species taxonomically related to *T. cacao* (Parmar et al., 2019) (**Figure 6**; **Supplementary Table 2**).

Additionally, RBOHD (Respiratory burst oxidase homolog D) was induced in both clones, suggesting that ABA signaling in *T. cacao*, is dependent on ROS production. This is consistent with reports of NADH-dependent, calcium-activated oxidase generating superoxide during stress responses (Kwak et al., 2006; Chapman et al., 2019) and with evidence linking RBOHD activity to lateral root development (Yao et al., 2017).

During the DS response, the transport of synthesized ABA to target organs is essential. In both cocoa clones, the genes ABCG11 and ABCG36 (ATP-binding cassette family G), homologous to AtABCG25 (a key ABA transporter), along with several NRT1/PTR family genes, were upregulated (**Figure 6**; **Supplementary Table 2**) (Shinozaki et al., 2017; Kuromori et al., 2018; Parmar et al., 2019). Other ABC transporters also showed differential expressions: ABCB20 in EET8, associated with auxin transport and detoxification, and ABCC3 in TSH565, linked to glutathione conjugate transport (Pierman et al., 2018).

Anion channels, which play critical roles in cell signaling, osmoregulation, metabolism, nutrition and stress tolerance were also implicated (Chen et al., 2019). In particular, the SLAC1 anion channel (Tc03v2_g022100), which mediates ABA-dependent stomatal closure by promoting anion and K^+^ efflux from guard cells to reduce turgor and minimize water loss was notably downregulated in EET8 (log_2_FC = -1.92) (Geiger et al., 2011). This downregulation suggests impaired stomatal regulation, potentially contributing to greater water loss, lower leaf water potential, reduced RWC in this clone (**Figure 7A**).

#### 4.2.3 Transcription factors involved in the response to DS

TFs are master regulators of gene expression (Osakabe et al., 2014; Yang et al., 2016). In EET8 and TSH565 clones, STOP1 (Tc09v2_g024900) and bHLH96 (Tc01v2_g008170), were overexpressed, while bZIP53 (Tc04v2_g024560) was specifically upregulated in TSH565. STOP1 regulates tolerance to multiple stresses including DS tolerance, hypoxia and heavy metal detoxification (Iuchi et al., 2007; Sadhukhan et al., 2017; Enomoto et al., 2019).

The bHLH TF family, one of the largest in plants, participates in developmental processes and stress signaling. They participate in jasmonic acid-mediated signaling pathways (Liu et al., 2014) and in ABA-mediated stomatal regulation through ABA kinases (AKs) substrates targeted by ABA-activated SNRK2 kinases (SNF1-related protein kinase 2s) during DS (Shinozaki et al., 2017). In maize, bHLH TF enhances drought tolerance by promoting root growth and ABA synthesis (Li et al., 2019). The strong upregulation of bHLH96 in both clones suggests a significant role in their drought response.

In TSH565, the TF bZIP53, a regulator of low energy signaling (**Supplementary Table 1**) was strongly upregulated by DS, potentially activating the asparagine biosynthesis pathway (ASN1, GLNS, ASP and PepCK) (Baena-González et al., 2007) (**Supplementary Table 2**). bZIP53 is also a target of SnRK1-like protein kinase (SNF1-related protein kinase 1), whose function is to regulate the energy balance that is essential for survival under stress (Dietrich et al., 2011). Interestingly, all these genes were upregulated in TSH565, counteracting the downregulation of other genes affecting energy metabolism under DS. These findings suggest that bZIP53 may help maintain energy homeostasis and contribute to enhanced DS tolerance observed in this clone.

Moreover, among the transcription factors induced in this clone, SRG1 (SCARECROW protein), which has been reported to be a repressor of the GA (gibberellic acid) signaling pathway, is involved in the negative control of plant growth (Yang et al., 2016).

Also, several TF were upregulated in EET8 clone indicating a strong and sustained activation of regulatory mechanisms mediated by multiple TF families. The maintenance of this transcriptional regulation at D52 suggests that the stress response in this clone is not transient but rather reflects a prolonged activation of regulatory pathways. This pattern is consistent with a strategy based on the continuous engagement of multiple signaling and regulatory mechanisms to cope with prolonged DS (Yang et al., 2016; Shinozaki et al., 2017).

#### 4.2.4 Membrane transport and aquaporin regulation under DS conditions in *T. cacao*

Similarly, the complex response to drought involves endomembrane trafficking, which is linked to signaling pathways through changes in cellular processes and cargo delivery (Wang et al., 2020b). In TSH565, the CNIH4 (Tc01v2_g031670) gene was highly upregulated under DS (**Supplementary Tables 1** and **5**). This gene encodes a cargo receptor involved in transporting integral membrane proteins to their proper locations (Rosas-Santiago et al., 2015, 2017). In *Arabidopsis*, CNIHs regulate the sorting, transport and localization of glutamate receptor channels (GLRs) that mediate Ca^2+^ transport in various contexts (Wudick et al., 2018). In rice, OsCNIH1, interacts with a sodium transporter to ensure its correct localization on the Golgi membrane (Aridor and Traub, 2002). The fact that CNIH4 upregulation occurred exclusively in clone TSH565 highlights the need to investigate its interaction with transported proteins to better understand its role in this clone´s response to DS.

Aquaporins (AQPs) are a family of transmembrane proteins that play a critical role in transporting water and other substrates, making them essential for plant stress responses. These proteins facilitate the bidirectional flow of water and solutes across cell membranes (Afzal et al., 2016; Sun et al., 2024). Among them, TIPs (Tonoplast Intrinsic Proteins) and PIPs (Plasma Membrane Intrinsic Proteins) primarily transport water by forming channels in the vacuole and plasma membranes regulating intracellular water flow and consequently cellular turgor (Małgorzata Kurowska, 2020). Interestingly, both clones showed AQP gene downregulation under DS, while in TSH565, the TIP3–2 gene (Tc03v2_g005540) was strongly upregulated (log_2_FC = 3.67) (**Supplementary Tables 2** and **5**). Interestingly, EET8 exhibited a greater reduction in RWC and ψ_leaf_, whereas TSH565, showed smaller reduction in these parameters along with an increase in WUE*_i_*. While AQP downregulation is a mechanism to reduce water loss during drought, their expression patterns can be highly variable (Małgorzata Kurowska, 2020). In some cases, AQPs are differentially regulated to redistribute water directly to critical tissues while limiting transport elsewhere (Merlaen et al., 2019). Overexpression of AQP genes in other species, such as tomato, resulted in high drought tolerance by improving the regulation of transpiration under stress (Sade et al., 2009).

These results suggest that differences in the regulation of AQP gene expression may underline the contrasting abilities of these clones to maintain cellular turgor and water flow within cells and tissues under DS.

#### 4.2.5 Metabolites and the antioxidant response to stress

Metabolites play a central role in plant drought responses, as genes involve in their biosynthesis are frequently upregulated under stress and correlate with their accumulation (Kumar et al., 2021; Li et al., 2024). In this study, several metabolic genes were highly upregulated in both clones under DS (**Supplementary Table 1**). Among them, GoIS, which catalyzes the first step of raffinose family oligosaccharide (RFO) biosynthesis, promotes the accumulation of galactinol and raffinose- osmoprotectants, that stabilize membranes, scavenge ROS, and protect the photosynthetic apparatus. Overexpression of GoIS2 has been linked with enhanced drought tolerance through increased endogenous levels of these metabolites (Taji et al., 2002; Xiao et al., 2023).

Similarly, P5CS, a key enzyme in proline biosynthesis and accumulation was induced in both TSH565 and EET8 (Kumar et al., 2021)(**Supplementary Table 2**). Proline contributes to osmotic adjustment, ROS scavenging, membrane integrity, and protein stabilization, thereby mitigating dehydration-induced damage (Ghosh et al., 2022). In *T. cacao*, proline accumulation has been linked to drought-tolerant genotypes displaying higher RWC and reduced stomatal opening under DS (Bae et al., 2009; Zakariyya et al., 2017; Dzandu et al., 2021). The expression profiles observed here suggest that both clones employ proline-mediated osmotic adjustment as part of their drought response.

Genes involved in the synthesis of additional compatible osmolytes, such as glycine-betaine (GB) and trehalose were also upregulated. These compounds protect thylakoid and plasma membrane, and help maintain photosynthetic efficiency under stress conditions (Basit et al., 2025) (**Supplementary Table 2**). Notably, TSH565 exhibited minimal alterations in photosynthetic machinery under DS, as reflected by stable F_v_/F_m_ values, smaller reduction in the ψ_leaf_ and a higher WUE*_i_*, whereas EET8 showed stronger reduction in these parameters. Although osmotic adjustment appears to be a common response in *T. cacao*, its activation in TSH565 may contribute more effectively to protecting the photosynthetic machinery from dehydration-induced damage.

Despite reductions in the ψ_leaf_ and F_v_/F_m_, the EET8 plants were able to withstand DS, potentially due to the strong upregulation of TPL (Thaumatin-like protein; Log_2_ FC = 9.2) (**Supplementary Table 1**). Thaumatin - like proteins, also referred to as osmotin-like proteins, are associated with tolerance to biotic, osmotic and drought stress and accumulate in plants adapted to dry environments (de Jesús-Pires et al., 2020; Faillace et al., 2021). Their overexpression in crops like sesame and tobacco enhances drought and salinity tolerance, improves survival under DS, and contributes to pathogen resistance through physiological, biochemical, and molecular adjustments (Rajam et al., 2007; Chowdhury et al., 2017). The marked induction of TPL in EET8 suggests a compensatory role in mitigating reductions in ψ_leaf,_ highlighting its potential as a candidate gene for improving crop tolerance through breeding strategies (Manghwar and Hussain, 2021).

Cell wall remodeling also appears to contribute to drought response. Expansins, which regulate cell growth and expansion under abiotic stress were induced in both clones (Krishnamurthy et al., 2019; Li et al., 2011) (**Supplementary Table 1**). In particular, EXLB1, previously associated with drought responses in *Brassica rapa*, was highly upregulated, suggesting its involvement in modulating cell wall dynamics in *T. cacao* under DS (Krishnamurthy et al., 2019).

In parallel, drought stress triggered a robust activation of antioxidant system, which is essential for limiting ROS-induced damage to cellular components and maintaining redox homeostasis (Noctor et al., 2014; Jain et al., 2018). Both clones, showed strong upregulation of genes such as MIOX, COMT, and PPOs (**Supplementary Table 1**). MIOX contributes to ascorbate biosynthesis and in rice has been linked to enhanced drought tolerance through improved ROS scavenging and proline accumulation (Duan et al., 2012; Shi et al., 2017). COMT, involved in melatonin biosynthesis, regulates stress responses, and its overexpression has been shown to enhance drought tolerance and recovery in *Arabidopsis* (Yang et al., 2019), while exogenous melatonin in *Chinese walnut* improved growth recovery, photosynthetic efficiency, ROS elimination, and proline accumulation (Sharma et al., 2020). Increased PPO activity under DS has also been reported in *T. cacao*, contributing to H_2_O_2_ detoxification as part of an adaptive stress mechanism (Santos et al., 2014).

Additional antioxidant related components exhibit clone-specific patterns. Peroxidase genes, along with glutathione S-transferases and aldehyde dehydrogenase, were predominantly induced in EET8, indicating a broad and coordinated oxidative stress response. In contrast, LEA proteins and sHSPs showed stronger upregulation in TSH565. These proteins enhance drought tolerance by stabilizing membranes and proteins, preventing aggregation, and facilitating protein refolding, suggesting complementary antioxidant strategies between clones (Wang et al., 2004; Ul Haq et al., 2019) (**Figure 6**; **Supplementary Table 2**).

Finally, KEGG pathway analysis revealed upregulation of genes involved in phenylpropanoid biosynthesis in both clones under DS, highlighting the importance of non-enzymatic antioxidant defenses. Activation of this pathway reported previously in *T. cacao* promotes the accumulation of phenolic compounds, which play critical roles in plant acclimation and adaptation (Osorio Zambrano et al., 2021). Due to their antioxidant activity these compounds scavenge ROS, mitigating protein oxidation and lipid peroxidation in cell membranes, avoiding the oxidative stress during drought stress (Sah et al., 2016; Ye et al., 2017). Together, these results underscore the coordinated contribution of enzymatic and nonenzymatic antioxidant systems in regulating intracellular ROS and sustaining cellular homeostasis during DS (Passardi et al., 2005; Mishra et al., 2023).

#### 4.2.6 Regulation of the photosynthetic apparatus during water deficit

In accordance with observed results at physiological level, DS induced changes in the expression of genes related to photosynthetic machinery. In TSH565, PsbP which encodes extrinsic PSII subunit involved in the normal oxidation of water molecules (Che et al., 2020) were upregulated (**Supplementary Table 2**). Studies in tobacco and *Arabidopsis* confirmed that PsbP is essential for PSII assembly, stabilization and photoautotrophy; loss-of-function mutants exhibit reduced growth, pale leaves and markedly lower F*_v_*/F*_m_* (0.47) (Ifuku et al., 2005; Yi et al., 2007). PsbP also contributes to stress tolerance under water deficit by regulating stomatal movement (Hong et al., 2020). The upregulation of these genes in TSH565 likely stabilized PSII during DS, and, together with GB-mediated osmoprotection, maintained photosynthetic function, suggesting that the lower reduction in A observed for this clone, was due to stomatal limitation rather than disruption of the photosynthetic machinery.

Conversely, in EET8, photosynthesis-related genes were downregulated (**Supplementary Table 2**), including PSBW, a core PSII component essential for structural stabilization and photosynthesis (Bishop et al., 2003; Soldatos et al., 2015). Loss of PSBW impairs PSII-LHCII complex formation, reducing energy transfer efficiency between PSII units and decreasing complex activity under excess light (García-Cerdán et al., 2011). In contrast, this gene was upregulated in drought-tolerant pearl millet clones submitted to drought (Dudhate et al., 2018).

Other photosynthetic genes were downregulated in EET8, (LHCs; PsaF; PsaH; Psak) that encodes PSI subunits, electron transport, and PSII subunits (**Supplementary Table 2**) indicating strongly repressed photosynthetic activity in this clone under DS. Proteomic and Transcriptomic studies in maize suggest that reduced LHC abundance under drought can serve as a protective strategy against photooxidative damage leading to reduced gas exchange and chlorophyll fluorescence (Dalal and Tripathy, 2018; Zhang et al., 2018). Therefore, the downregulation of these genes observed in EET8 may reflect a protective mechanism against the oxidative damage of its photosynthetic machinery.

### 4.3 Transcriptomic and physiological data integration

To integrate transcriptomic profiling with physiological and growth variables, a Multiple Factor Analysis (MFA) was realized. The MFA successfully integrated our transcriptomic with physiological and growth data, with the first dimension (30.9% variance) separating plants by water treatment, and confirming a wholesale shift in the integrated phenotype under stress. Within the stressed plants, the analysis revealed a critical divergence along a dimension 2 (17.2% variance), which we interpret as a gradient of physiological tolerance (**Supplementary Figure 8**). This gradient clearly distinguished TSH565 clone—characterized by superior maintenance of photosynthesis (A, Fv/Fm), stomatal conductance (g*s*), water-use efficiency (WUE*i*) and total dry biomass accumulation (DWTo)—from the more impaired EET8 clone. The alignment of this physiological gradient with the major transcriptomic principal components suggests that TSH565 resilience is facilitated by a distinct and effective transcriptional strategy, potentially more targeted or efficient than the broader stress-response program observed in EET8, as revealed by the ORA of GOterms previously described. This integrated view moves beyond simple gene expression differences or signatures to pinpoint the functional coupling between complex molecular reprogramming and physiological outcome as a key determinant of performance under DS.

## 5 Concluding remarks

The physiological and transcriptomic responses of *T. cacao* clones to DS were congruent, indicating a coordinated molecular reprogramming of gene expression profiles directed to cellular protection, metabolite synthesis and accumulation, and allowing recovery from prolonged stress. This response involved not only genes with metabolic or physiological functions but also those related to signal transduction and gene regulation, including transcription factors associated with drought tolerance (**Figure 6**).

While this integrated analysis confirmed a genotype−specific divergence under stress, the main added value behind is the identification of robust candidate genes. The candidate genes identified and proposed in this study, form part of the molecular framework behind the contrasting responses observed, driving to drought tolerance in both cacao clones (**Supplementary Table 1**). This reinforces that drought resilience in cacao is mediated by integrated multi-gene regulatory networks and provides a biologically informed starting point for future functional validation and breeding efforts aimed at understanding and improving drought tolerance in cacao.

In TSH565, photosynthesis was limited by stomatal closure due to DS, but with moderate impairment the photosynthetic machinery. Upregulation of genes involved in osmoprotectant synthesis, antioxidant systems, compatible osmolytes and aquaporins helped to maintain intracellular water flow and cellular turgor, reflected in higher water use efficiency, enhanced root growth and maximal water absorption. Signaling occurred through both ABA-dependent and -independent pathway, collectively supporting better physiological performance and reduced impact from DS.

In contrast, EET8 experienced both stomatal and nonstomatal limitations, indicating partial damage to the photosynthetic apparatus. Although this clone also activated antioxidant systems, transcription factors, and osmotic adjustment mechanisms like THS565, it exhibited lower efficiency in maintaining water balance and higher stomatal restrictions to limit water loss. The response was more pronounced, involving a greater number and diversity of functional categories and metabolic pathways, including cross talk with other biotic and abiotic stresses, and known mechanisms of cell oxidative damage repair. Together, these patterns are likely to reflect a higher level of stress and a greater disruption of cellular homeostasis, suggesting multiple strategies were mobilized to cope with DS. Despite the higher energetic cost, these mechanisms enabled recovery seven days after rehydration, demonstrating a tolerance phenotype.

Integration of transcriptomic and physiological data in the MFA, which clearly segregated the two genotypes under stress conditions, confirmed this fundamental trade-off in drought response strategies among these two clones (**Supplementary Figure 8**), suggesting that the divergences at the transcriptomic and physiological level are clearly but complexly correlated (**Supplementary Tables 10**-**14**).These findings highlight that while EET8 and TSH565 clones showed both drought-tolerance traits, the underlying molecular and physiological mechanisms and strategy substantially differ. Characterizing this variability, seemingly genotype-dependent, is essential, as these diverse mechanisms may be preserved in germplasm banks and implemented in breeding programs. Functional genomics approaches, such as RNA-seq integrated with physiological evaluations, are valuable for dissecting polygenic traits.

This study provides new insights into the molecular bases of drought stress tolerance in *T. cacao* and identifies candidate genes for future research aimed at enabling genetic adaptation to environmental challenges, including those posed by global warming.

## Data Availability

The datasets presented in this study can be found in online repositories. The names of the repository/repositories and accession number(s) can be found below: https://www.ebi.ac.uk/arrayexpress/, E-MTAB-8525 https://www.ebi.ac.uk/ena, ERP118455.
