# Supplementary material for: All roads lead to Rome: integrated physiological and transcriptomic analysis of cacao drought response reveals different ways to achieve tolerance in two hybrid clones
Source: Front Plant Sci. 2026 Feb 18;17:1764400. doi: 10.3389/fpls.2026.1764400 (PMC12957243; doi:10.3389/fpls.2026.1764400)
Supplement: Supplementary file 1 [file DataSheet1.pdf]

Supplementary Figures

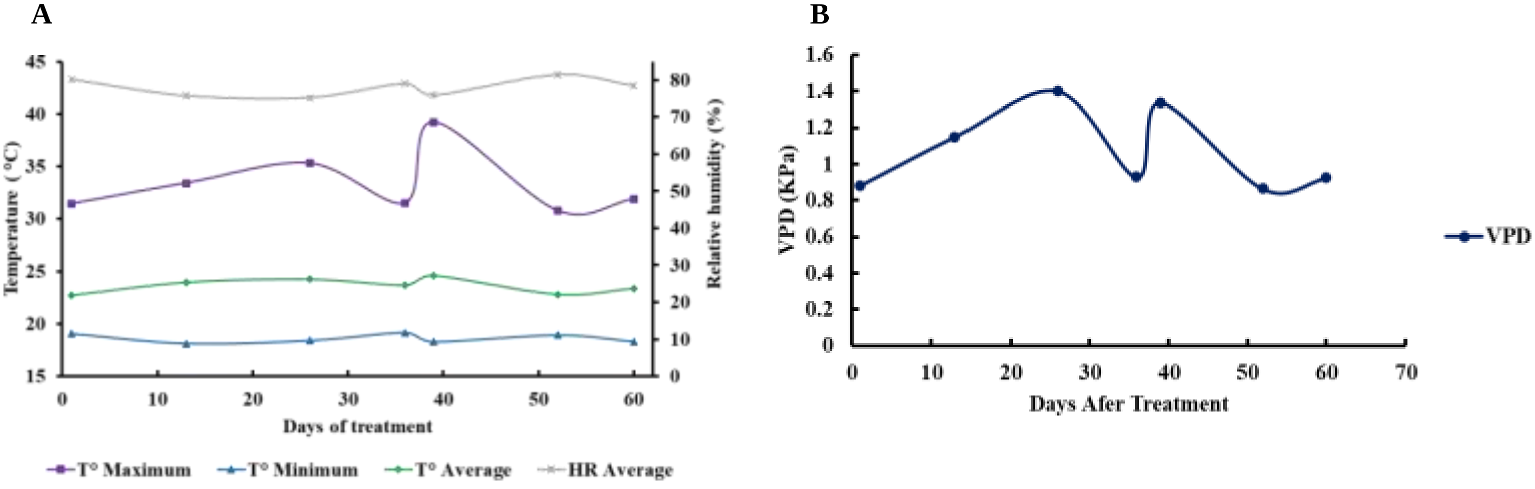

Figure S1. Variation of the conditions of (**A**) Daily mean temperature and relative humidity; and (**B**) Vapor pressure deficit (VPD) in the greenhouse during the period of water deficit and recovery

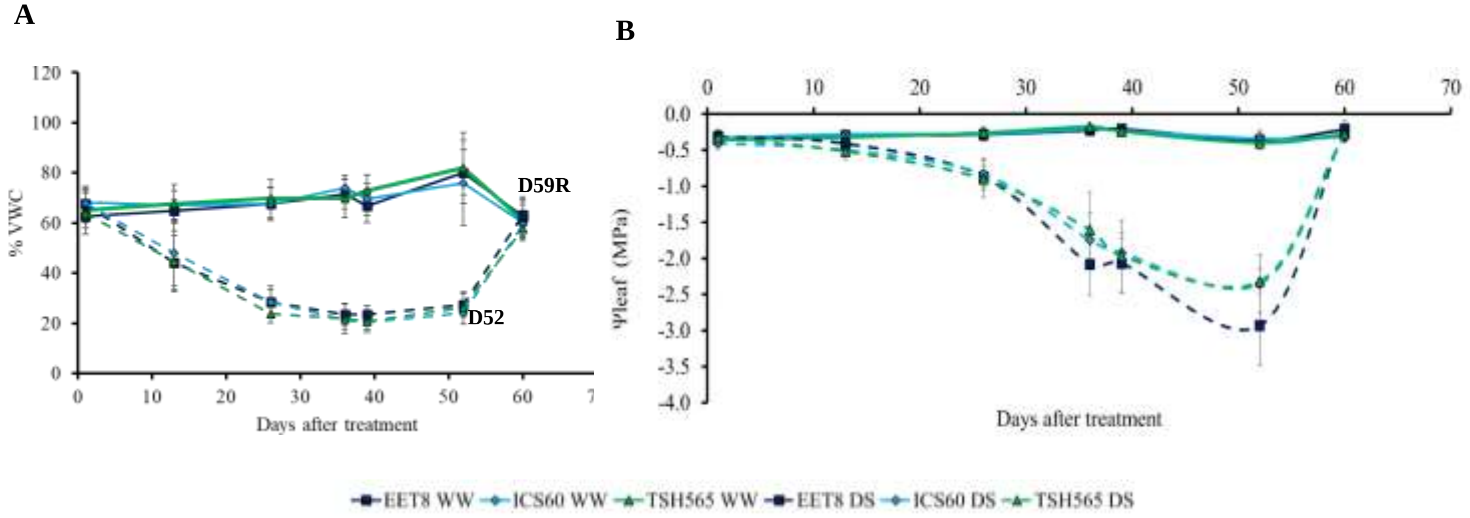

Figure S2. **Variation in (A)** soil volumetric water content (VWC) and **(B)** predawn leaf water potential (Ψ_{leaf}) of the cacao clones EET8, ICS60 and TSH565 under the two water status conditions. WW, plants watered to field capacity (solid lines) and DS, plants subjected to water deficit (dashed lines). Values are mean ± SD (n = 6). The day of maximum stress (D52) and the day of measurements after rehydration (D59R) are indicated.

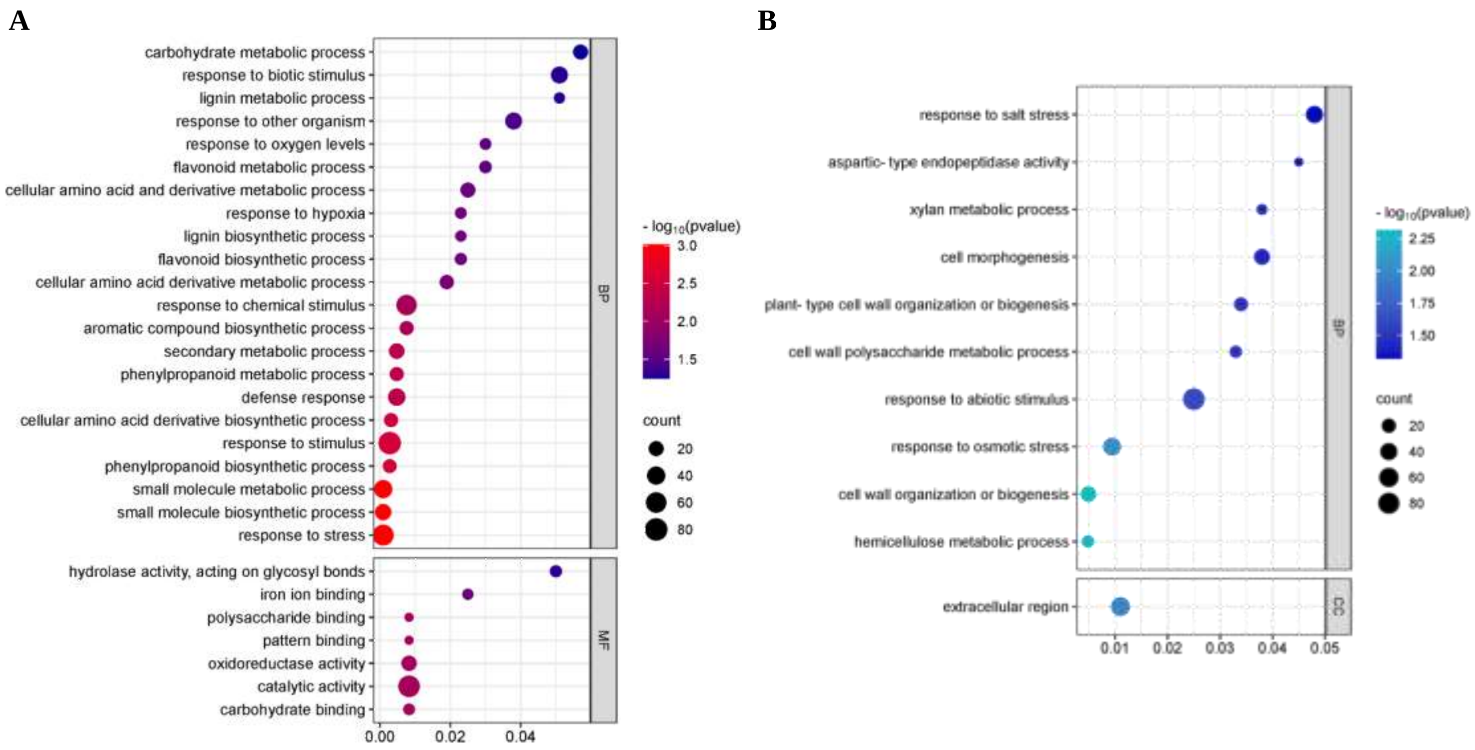

Figure S3. Main GO categories represented in the common DEGs with the highest expression changes (log2FC ≥ 2 or ≤ −2) between the clones EET8 and TSH565 in response to water deficit stress. (**A**) Common upregulated genes. (**B**) Common downregulated genes

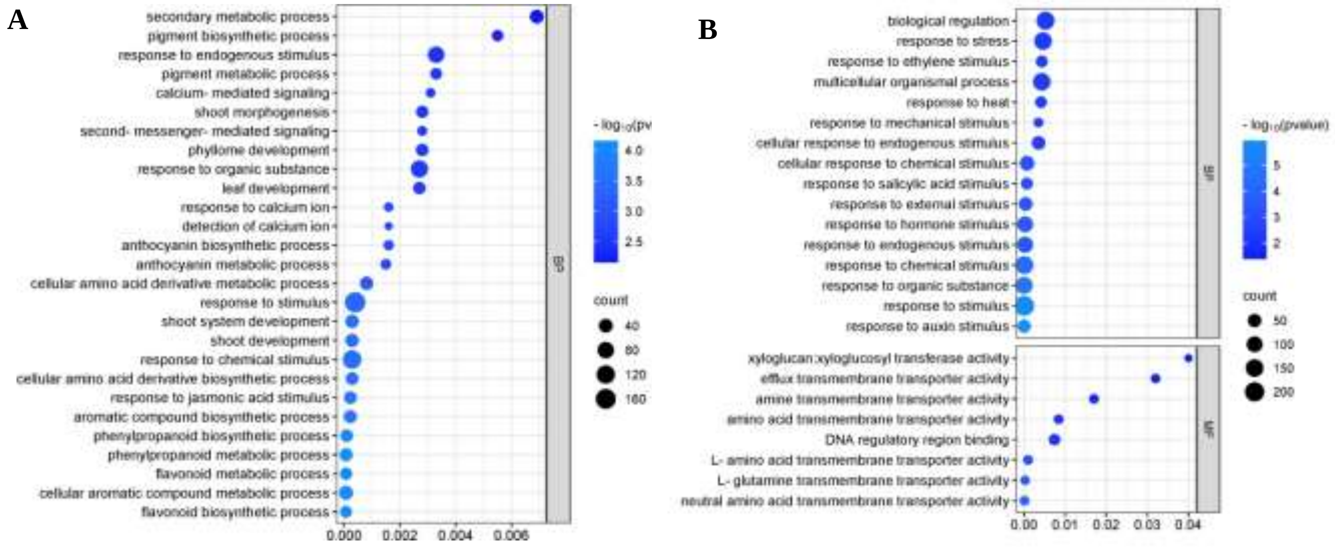

Figure S4. Main GO categories represented in the clone-specific downregulated DEGs (log2FC ≤ −2) of EET8 and TSH565 in response to water deficit stress. (**A**) Genes downregulated in TSH565 in response to DS. (**B**) Genes downregulated in EET8 in response to DS.

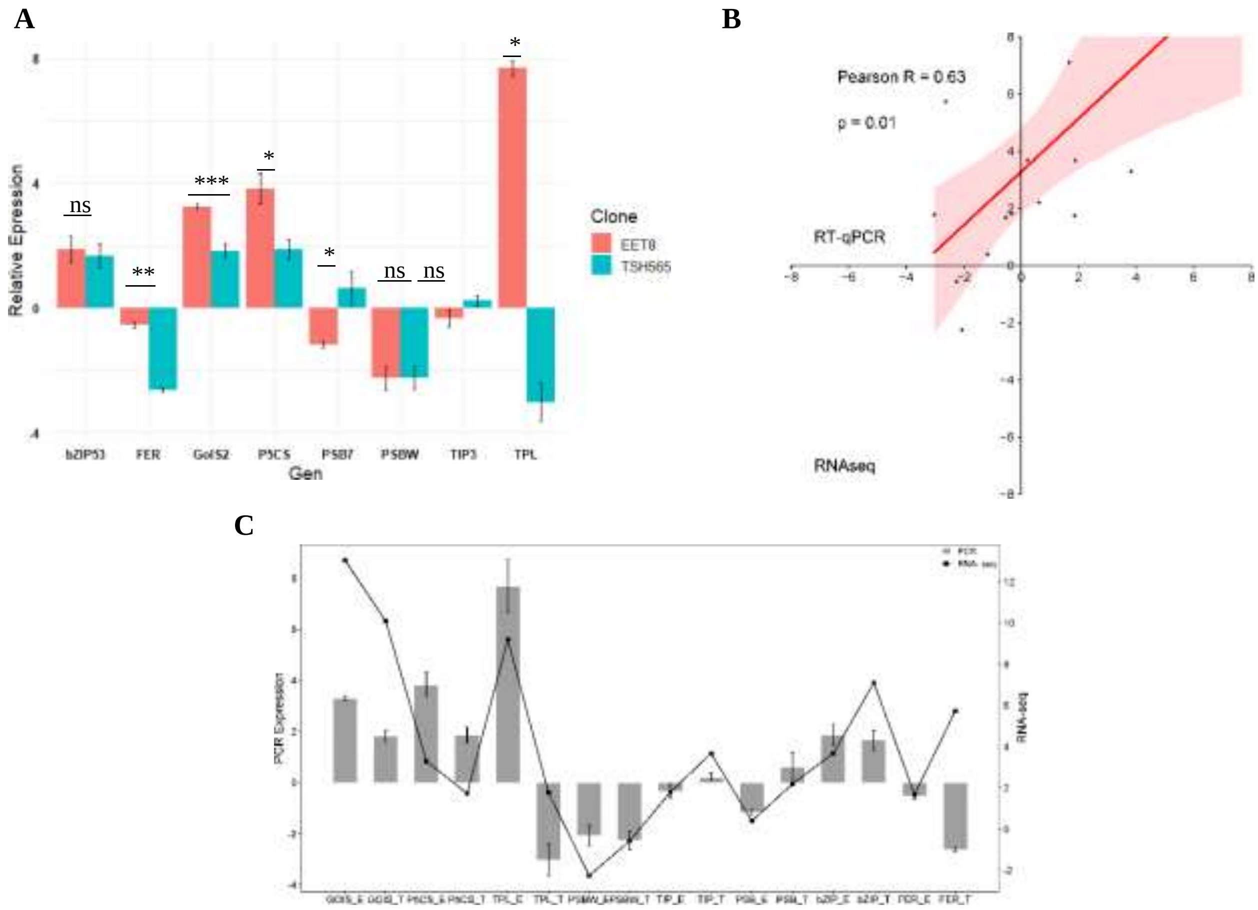

Figure S5. Validation of RNA-seq results by RT-qPCR. (**A**) Quantitative reverse transcription PCR (RT-qPCR) validation of eight differentially expressed genes (DEGs) in EET8 and TSH565 clones after drought treatment. Relative expression levels were calculated by normalizing DEG expression to the UBQ reference gene. Bars represent mean ± SE; (n = 5). Asterisks indicate significance levels ($p < 0.05$, $p < 0.01$, $p < 0.001$). A *t*-test was applied when data met normality assumptions (Shapiro–Wilk test), and the Wilcoxon test was used otherwise. The analyzed genes were: bZIP53, putative bZIP transcription factor 53 (Tc04v2_g024560); FER, putative receptor-like protein kinase FERONIA (Tc02v2_g028060); GolS2, galactinol synthase 2 (Tc04v2_g025290); P5CS, Δ^1-pyrroline-5-carboxylate synthase (Tc05v2_g002790); PSBP, PsbP domain-containing protein 7, chloroplastic (Tc05v2_g02577); PSBW, photosystem II reaction center W protein, chloroplastic (Tc05v2_g023360); TIP3, probable aquaporin TIP3-2 (Tc03v2_g005540); and TPL, thaumatin-like protein (Tc03v2_g022930).

(B) Pearson correlation between RNA-seq and RT-qPCR expression values.

(C) Comparison of RNA-seq and RT-qPCR expression profiles of the eight selected genes in both cacao clones. Bar plots represent RT-qPCR data, and line plots represent RNA-seq data. Gene names are shown with clone initials, where “E” corresponds to EET8 and “T” corresponds to TSH565 (e.g., GolS2_E and GolS2_T).

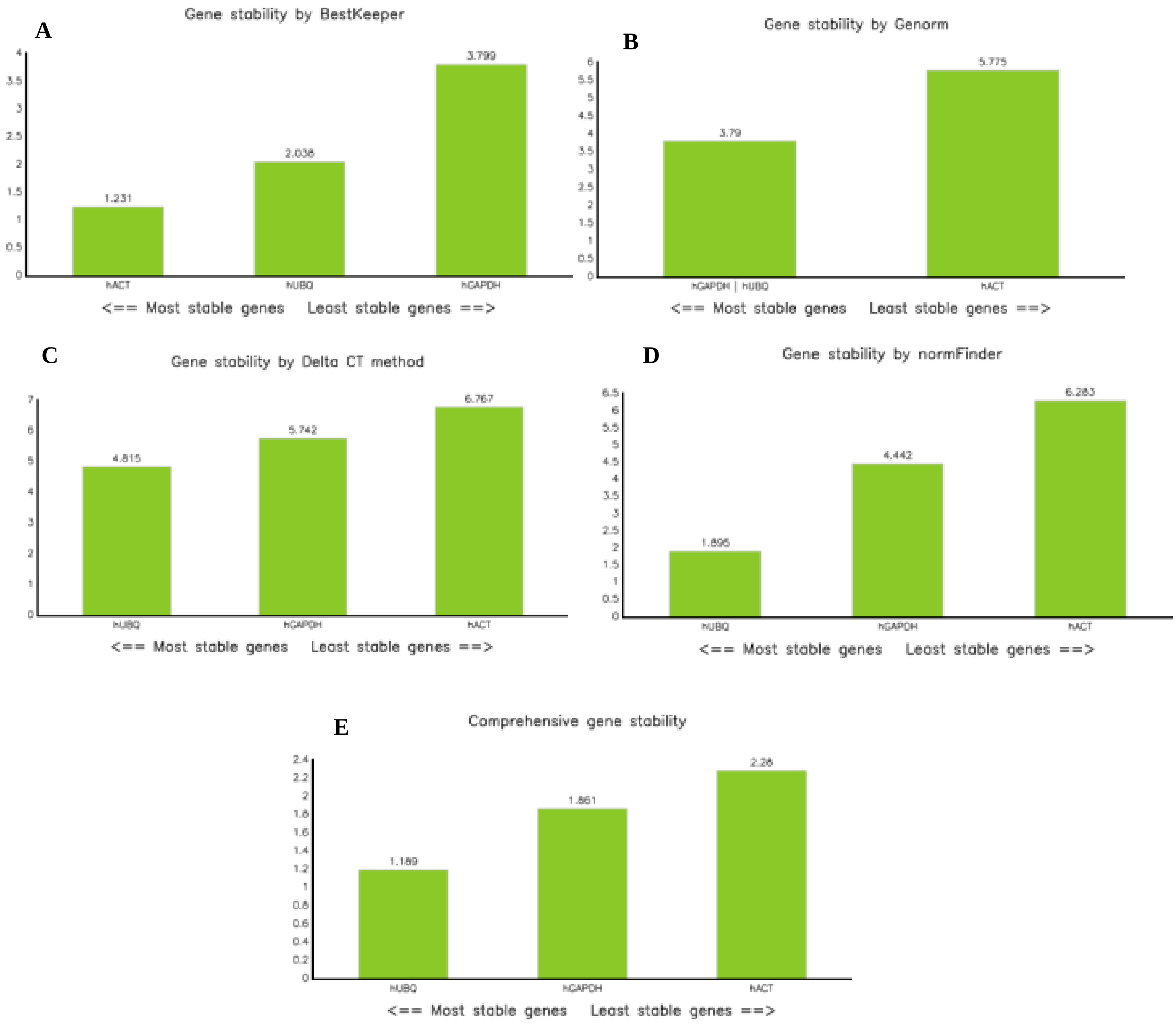

Figure S6. Stability ranking obtained using the RefFinder online tool integrating geNorm, NormFinder, BestKeeper and ΔCt methods. (**A**) Gene stability based on bestkeeper algorithm; (**B**) Gene stability based on Genorm algorithm; (**C**) Gene stability based on Delta CT method; (**D**) Gene stability based on normFinder algorithm; (**E**) Ranking of comprehensive gene stability generated by RefFinder based on the four statistical algorithms (A, B, C y D).

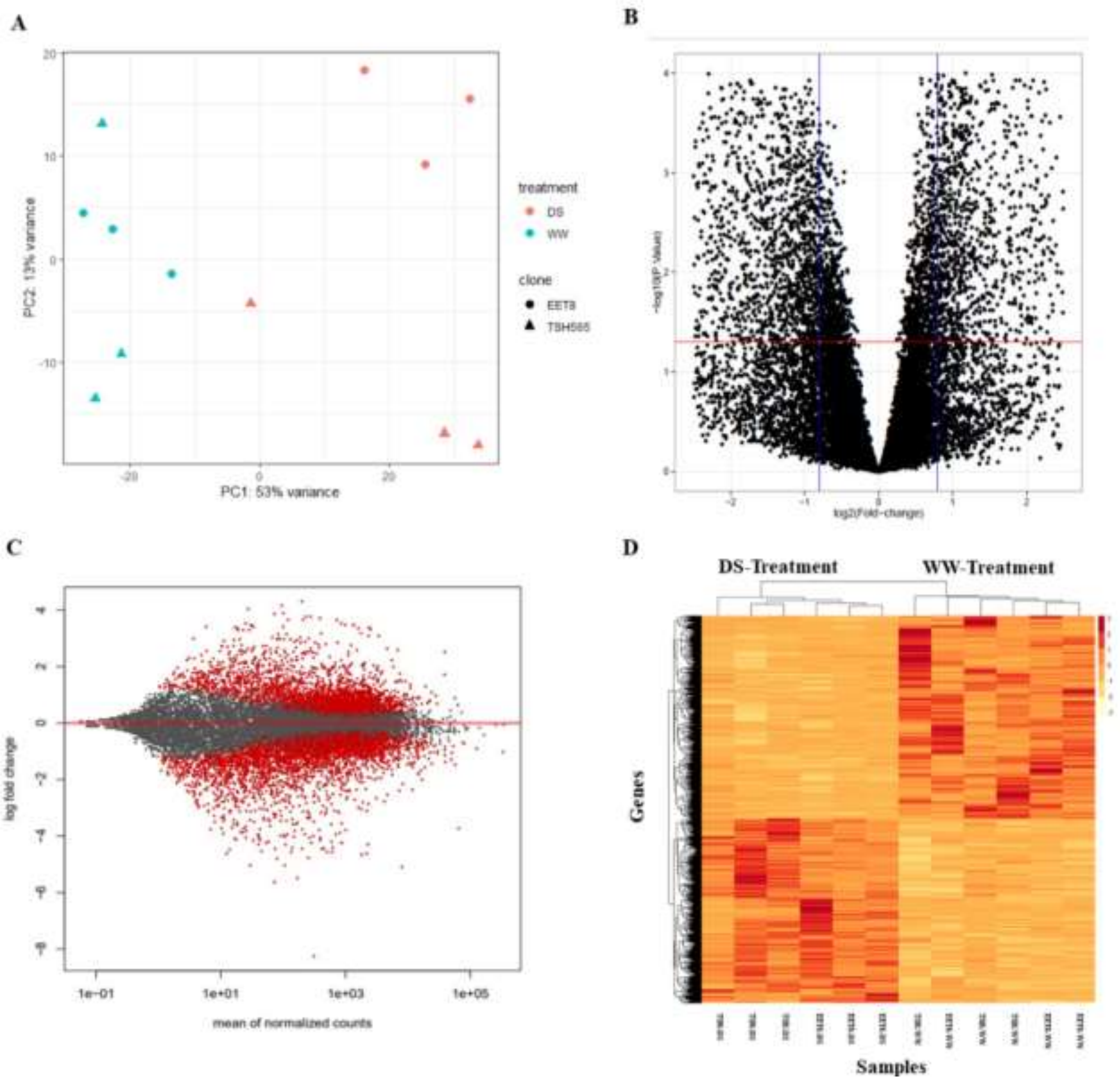

Figure S7. **Global Gen Expression Analysis of RNAseq data.** (**A**) Principal Component Analysis (PCA) clustering biological replicates and separation between treatment based on normalized gene expression profiles. (**B**) Volcano Plot illustrates the distribution of DEGs genes according to statistical significance P adjust ≤ 0.05 and magnitude of change log2FC ≥ 1. (**C**) MA-plot showing relationship between mean expression and fold chance for all detected genes. Red dots indicate significant DEGs (P adjust ≤ 0.05). (**D**) Heat map with expression profiles of significant DEGs. Rows correspond to genes and columns to samples. Color intensity ranges from light orange (downregulation) to dark orange (upregulation).

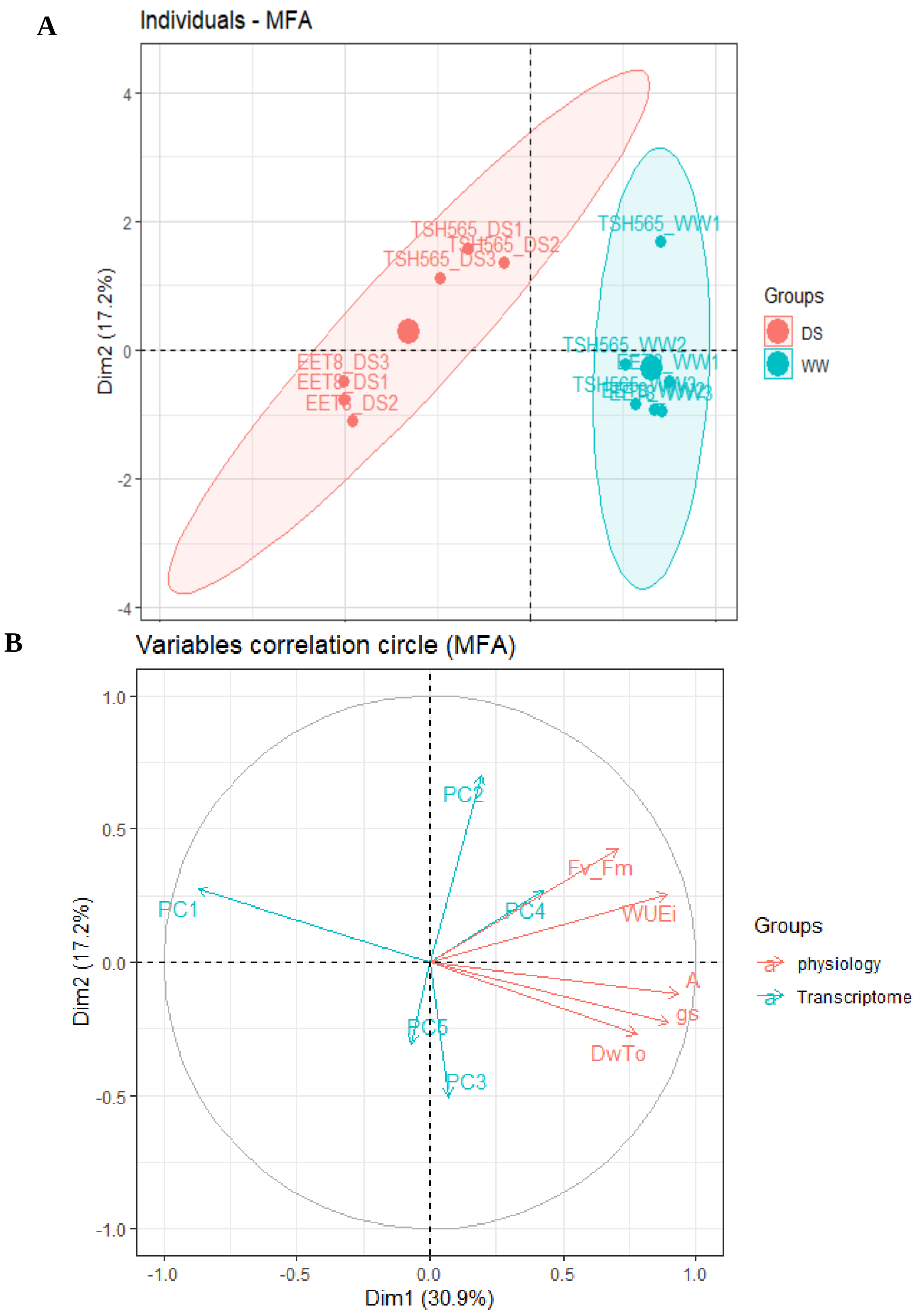

Figure S8. **Multiple Factor Analysis (MFA) integrating transcriptomic and physiological data. (A)** Factor map of individuals projected onto the first two MFA dimensions (Dim1 and Dim2). Samples are colored according to treatment (DS and WW), labels indicate clone identity and ellipses represent group dispersion. (**B**) Correlation circle of variables showing transcriptomic first five principal components (PC1-PC5), with physiological and growth variables (A,g_s,WUE$_i$, F_v/F_m, DwTo). Arrow length and direction indicate the strength and sign of correlation with MFA dimensions. Colors indicate the different data blocks (Transcriptomic PCs and physiological data). The MFA was performed using principal component scores derived from transcriptomic PCs explaining 83% of the total variance of transcriptomic data integrated with physiological datasets as two data blocks.
